# Supplementary material for: Chemical Constituents of the Marine-Derived Fungus Aspergillus sp. SCS-KFD66
Source: Mar Drugs. 2018 Nov 26;16(12):468. doi: 10.3390/md16120468 (PMC6316597; doi:10.3390/md16120468)

Chemical Constituents of the Marine-Derived Fungus *Aspergillus* sp. SCS-KFD66

Chang-Liang An [1,2,†], Fan-Dong Kong [1,†], Qing-Yun Ma [1], Qing-Yi Xie [1], Jing-Zhe Yuan [1], Li-Man Zhou[1], Hao-Fu Dai [1], Zhi-Fang Yu [2,*], and You-Xing Zhao [1,*]

1 Hainan Key Laboratory for Research and Development of Natural Product from Li Folk Medicine, Institute of Tropical Bioscience and Biotechnology, Chinese Academy of Tropical Agricultural Sciences, Haikou 571101, China; annncl@163.com (C.A.); kongfandong@itbb.org.cn (F.K.); maqingyun@itbb.org.cn (Q.M.); xieqingyi@itbb.org.cn (Q.X.); jingzhe1989@yahoo.com (J.Y.); zhouliman88@126.com (L.Z.); daihaofu@itbb.org.cn (H.D.)

2 College of Food Science and Technology, Nanjing Agricultural University, Nanjing 210095, China

* Correspondence: yuzhifang@njau.edu.cn (Z.Y.); zhaoyouxing@itbb.org.cn (Y.Z.); Tel.: +86-139-5169-2350 (Z.Y.); +86-898-66989095 (Y.Z.)

† These authors contributed equally to this paper

Received: 5 November 2018; Accepted: 21 November 2018; Published: date

Abstract: Five new compounds named asperpenes A-C (**1–3**), 12,13-dedihydroversiol (**4**), and methyl 6-oxo-3,6-dihydro-2*H*-pyran-4-carboxylate (**5**), along with 10 known compounds (**6–15**), were isolated from the fermentation broth of *Aspergillus* sp. SCS-KFD66 associated with a bivalve mollusk, *Sanguinolaria chinensis*, collected from Haikou Bay, China. The structures of the compounds, including the absolute configurations of their stereogenic carbons, were unambiguously determined by spectroscopic data, single-crystal X-ray diffraction analysis, and electronic circular dichroism (ECD) spectral analysis, along with quantum ECD calculations. The growth inhibitory activity of the compounds against four pathogenic bacterial (*Escherichia coli* ATCC 25922, *Staphylococcus aureus* ATCC 6538, *Listeria monocytogenes* ATCC 1911, and *Bacillus subtilis* ATCC 6633), their enzyme inhibitory activities against acetylcholinesterase and α-glucosidase, and their DPPH radical scavenging activity were evaluated.

Keywords: marine-derived fungus; *Aspergillus* sp.; secondary metabolites; antibacterial activity

List of Supporting Information

The 18S gene sequences of *Aspergillus* sp. SCS-KFD66……..……….…...………...……………...4

Theory and Calculation Details………...………...………....……….....……….....……………....5

Figure S1. The ^{1}H-NMR spectrum of compound **1** in CD_3OD………...………....………...……6

Figure S2. The ^{13}C-NMR spectrum of compound **1** in CD_3OD………...………....………...……7

Figure S3. The DEPT spectrum of compound **1** in CD_3OD………...………....………...…........8

Figure S4. The HMQC spectrum of compound **1** in CD_3OD………...………...………...………9

Figure S5. The ^{1}H-^{1}H COSY spectrum of compound **1** in CD_3OD ………...………....………..…10

Figure S6. The HMBC spectrum of compound **1** in CD_3OD………...………....………...……11

Figure S7. The ROESY spectrum of compound **1** in CD_3OD………...………...………....……12

Figure S8. The HRESIMS and IR spectra of compound **1**………...………...………...………13

Figure S9. The ^{1}H-NMR spectrum of compound **2** in $CDCl_3$………...………...………...……14

Figure S10. The ^{13}C-NMR spectrum of compound **2** in $CDCl_3$………...………...………...….15

Figure S11. The DEPT spectrum of compound **2** in $CDCl_3$………...……….…………...………16

Figure S12. The HMQC spectrum of compound **2** in $CDCl_3$………...………...………....……17

Figure S13. The ^{1}H-^{1}H COSY spectrum of compound **2** in $CDCl_3$ ………...………....…………18

Figure S14. The HMBC spectrum of compound **2** in $CDCl_3$………...………...………...……19

Figure S15. The ROESY spectrum of compound **2** in $CDCl_3$………...………...………...……20

Figure S16. The HRESIMS and IR spectra of compound **2**………...………….………...………21

Figure S17. The ^{1}H-NMR spectrum of compound **3** in $CDCl_3$………...………....………...……22

Figure S18. The ^{13}C-NMR spectrum of compound **3** in $CDCl_3$………...……….……...………23

Figure S19. The DEPT spectrum of compound **3** in $CDCl_3$………...………...………...………24

Figure S20. The HMQC spectrum of compound **3** in $CDCl_3$………...………...………....……25

Figure S21. The ^{1}H-^{1}H COSY spectrum of compound **3** in $CDCl_3$………...……….....…………26

Figure S22. The HMBC spectrum of compound **3** in $CDCl_3$………...………...………...………27

Figure S23. The ROESY spectrum of compound **3** in $CDCl_3$………...………...………...………28

Figure S24. The HRESIMS and IR spectra of compound **3**………...………...………...………29

Figure S25. The ^{1}H-NMR spectrum of compound **4** in $CDCl_3$……...………...………...………30

Figure S26. The ^{13}C-NMR spectrum of compound **4** in $CDCl_3$………...……….....…...………31

Figure S27. The DEPT spectrum of compound **4** in $CDCl_3$………...………...………...………32

Figure S28. The HMQC spectrum of compound **4** in $CDCl_3$………...………...………...……33

Figure S29. The ^{1}H-^{1}H COSY spectrum of compound **4** in $CDCl_3$………...………....…………34

Figure S30. The HMBC spectrum of compound **4** in $CDCl_3$………...………...………...………35

Figure S31. The ROESY spectrum of compound **4** in $CDCl_3$………...………...………...……36

Figure S32. The HRESIMS and IR spectra of compound **4**………...………...………...………37

Figure S23. The ^{1}H-NMR spectrum of compound **5** in $CDCl_3$……….....………....………...…….38

Figure S34. The ^{13}C-NMR spectrum of compound **5** in $CDCl_3$……….....………....………..…….39

Figure S35. The DEPT spectrum of compound **5** in $CDCl_3$……….....………...………....……….40

Figure S36. The HMQC spectrum of compound **5** in $CDCl_3$. ……….....………....………....…….41

Figure S37. The ^{1}H-^{1}H COSY spectrum of compound **5** in $CDCl_3$……….....………....………….42

Figure S38. The HMBC spectrum of compound **5** in $CDCl_3$……….....………....……....……….43

Figure S39. The HRESIMS and IR spectra of compound **5**……….....………....………....……….44

18S gene sequences of *Aspergillus* sp. SCS-KFD66

CATTAAATCAGTTATCGTTTATTTGATAGTACCTTACTACATGGATACCTGTGGTAATT
CTAGAGCTAATACATGCTAAAAACCTCGACTTCGGAAGGGGTGTATTTATTAGATAA
AAAACCAATGCCCTTCGGGGCTCCTTGGTGAATCATAATAACTTAACGAATCGCATG
GCCTTGCGCCGGCGATGGTTCATTCAAATTTCTGCCCTATCAACTTTCGATGGTAGGAT
AGTGGCCTACCATGGTGGCAACGGGTAACGGGGAATTAGGGTTCGATTCCGGAGAGG
GAGCCTGAGAAACGGCTACCACATCCAAGGAAGGCAGCAGGCGCGCAAATTACCCA
ATCCCGATACGGGGAGGTAGTGACAATAAATACTGATACGGGGCTCTTTTGGGTCTC
GTAATTGGAATGAGTACAATTTAAATACCTTAACGAGGAACAATTGGAGGGCAAGTC
TGGTGCCAGCAGCCGCGGTAATTCCAGCTCCAATAGCGTATATTAAAGTTGTTGCAGT
TAAAAAGCTCGTAGTTGAACCTTGGGTCTGGCTGGCCGGTCCGCCTCACCGCGAGTA
CTGGTCCGGCTGGACCTTTCCTTCTGGGGAACCTCATGGCCTTCACTGGCTGTGGGGG
GAACCAGGACTTTTACTGTGAAAAAATTAGAGTGTTCAAAGCAGGCCTTTGCTCGAA
TACATTAGCATGGAATAATAGAATAGGACGTGCGGTTCTATTTTGTTGGTTTCTAGGA
CCGCCGTAATGATTAATAGGGATAGTCGGGGGCGTCAGTATTCAGCTGTCAGAGGTG
AAATTCTTGGATTTGCTGAAGACTAACTACTGCGAAAGCATTCGCCAAGGATGTTTTC
ATTAATCAGGGAACGAAAGTTAGGGGATCGAAGACGATCAGATACCGTCGTAGTCTT
AACCATAAACTATGCCGACTAGGGATCGGGCGGTGTTTCTATGATGACCCGCTCGGC
ACCTTACGAGAAATCAAAGTTTTTGGGTTCTGGGGGGAGTATGGTCGCAAGGCTGAA
ACTTAAAGAAATTGACGGAAGGGCACCACAAGGCGTGGAGCCTGCGGCTTAATTTGA
CTCAACACGGGGAAACTCACCAGGTCCAGACAAAATAAGGATTGACAGATTGAGAG
CTCTTTCTTGATCTTTTGGATGGTGGTGCATGGCCGTTCTTAGTTGGTGGAGTGATTTGT
CTGCTTAATTGCGATAACGAACGAGACCTCGGCCCTTAAATAGCCCAAGA

Theory and Calculation Details. The calculations were performed by using the density functional theory (DFT) as carried out in the Gaussian 03.[S1] The preliminary conformational distributions search was performed by HyperChem 7.5 software. All ground-state geometries were further optimized at the B3LYP/6-31G(d) level. Conformers within a 2 kcal/mol energy threshold from the global minimum were selected to calculate the electronic transitions [S2]. The overall theoretical ECD spectra were obtained according to the Boltzmann weighting of each conformers. Solvent effects of methanol solution were evaluated at the same DFT level by using the SCRF/PCM method. [S3]

(S1) Gaussian 03, Revision E.01, M. J. Frisch, G. W. Trucks, H. B. Schlegel, G. E. Scuseria, M. A. Robb, J. R. Cheeseman, J. A. Montgomery, Jr., T. Vreven, K. N. Kudin, J.C. Burant, J. M. Millam, S. S. Iyengar, J. Tomasi, V. Barone, B. Mennucci, M. Cossi, G. Scalmani, N. Rega, G. A. Petersson, H. Nakatsuji, M. Hada, M. Ehara, K. Toyota, R. Fukuda, J. Hasegawa, M. Ishida, T. Nakajima, Y. Honda, O. Kitao, H. Nakai, M. Klene, X. Li, J. E. Knox, H. P. Hratchian, J. B. Cross, V. Bakken, C. Adamo, J. Jaramillo, R. Gomperts, R. E. Stratmann, O. Yazyev, A. J. Austin, R. Cammi, C. Pomelli, J. W. Ochterski, P. Y. Ayala, K. Morokuma, G. A. Voth, P. Salvador, J. J. Dannenberg, V. G. Zakrzewski, S. Dapprich, A. D. Daniels, M. C. Strain, O. Farkas, D. K. Malick, A. D. Rabuck, K. Raghavachari, J. B. Foresman, J. V. Ortiz, Q. Cui, A. G. Baboul, S. Clifford, J. Cioslowski, B. B. Stefanov, G. Liu, A. Liashenko, P. Piskorz, I. Komaromi, R. L. Martin, D.J. Fox, T. Keith, M. A. Al-Laham, C. Y. Peng, A. Nanayakkara, M. Challacombe, P. M. W. Gill, B. Johnson, W. Chen, M. W. Wong, C. Gonzalez, and J. A. Pople, Gaussian, Inc., Wallingford CT, 2004.

(S2) Sai, C.; Li, D.; Xue, C.; Wang, K.; Hu, P.; Pei, Y.; Bai, J.; Jing Y.; Li, Z.; Hua H. *Org. Lett.* **2015**, *17*, 4102-5.

(S3) (a) Miertus, S.; Tomasi, *J. Chem. Phys.* **1982**, *65*, 239–245. (b) Tomasi, J.; Persico, M. *Chem.Rev.* **1994**, *94*, 2027–2094. (c) Cammi, R.; Tomasi, J. J. *Comp.Chem.* **1995**, *16*, 1449–1458.

Figure S1. The ^1H NMR spectrum of compound **1** in CD_3OD

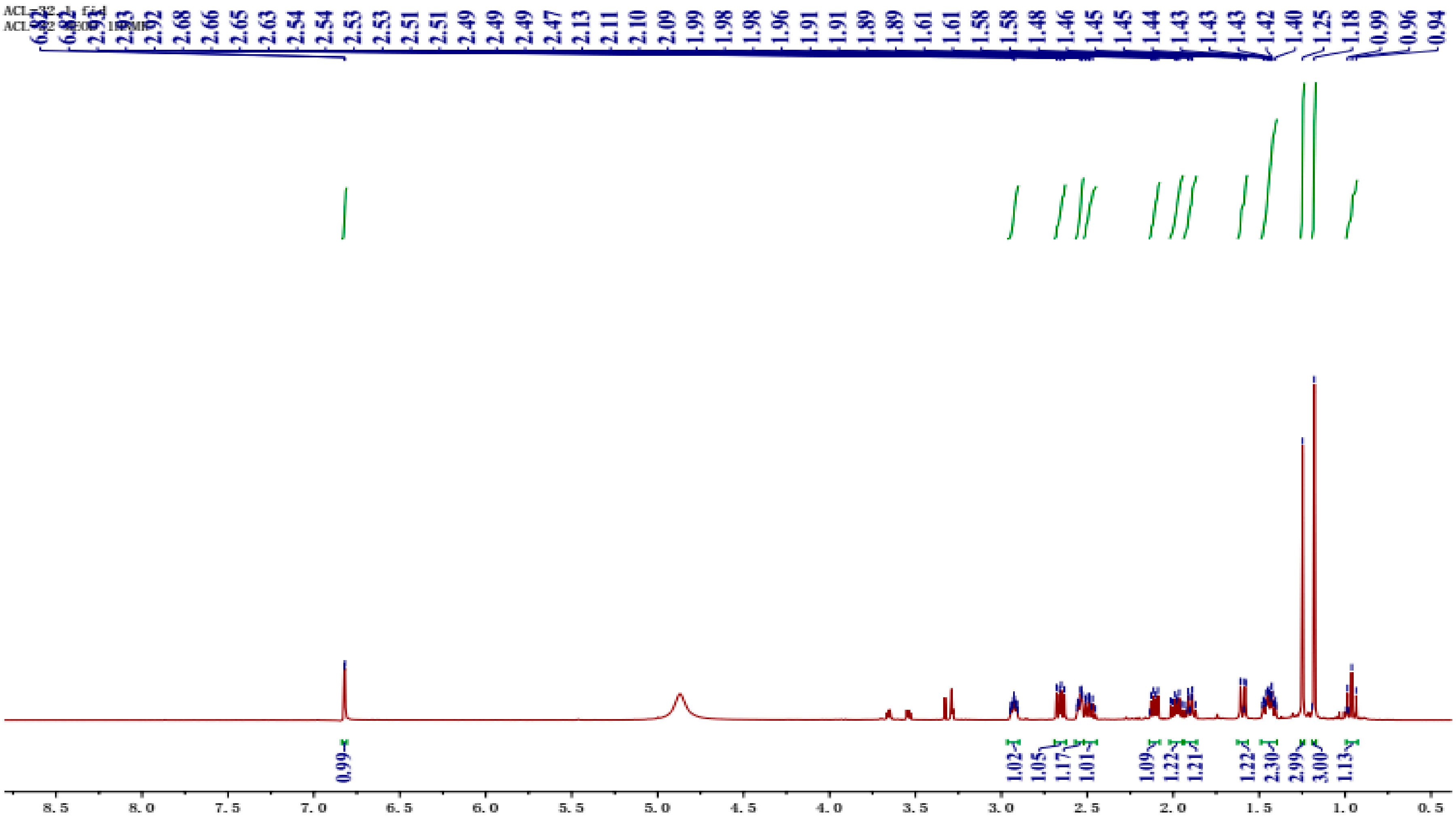

Figure S2. The ^{13}C-NMR spectrum of compound **1** in CD_3OD

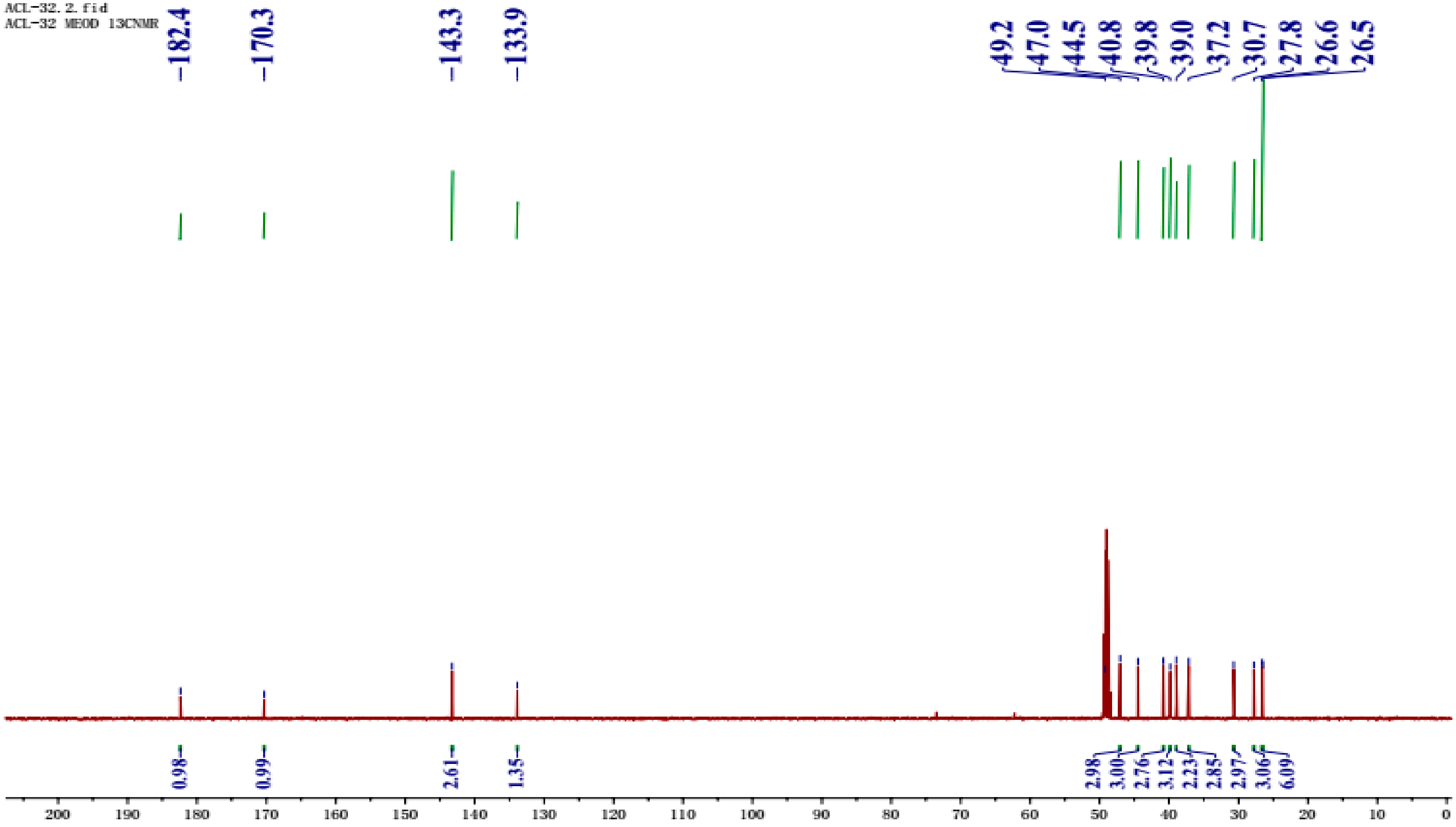

Figure S3. The DEPT spectrum of compound **1** in CD_3OD

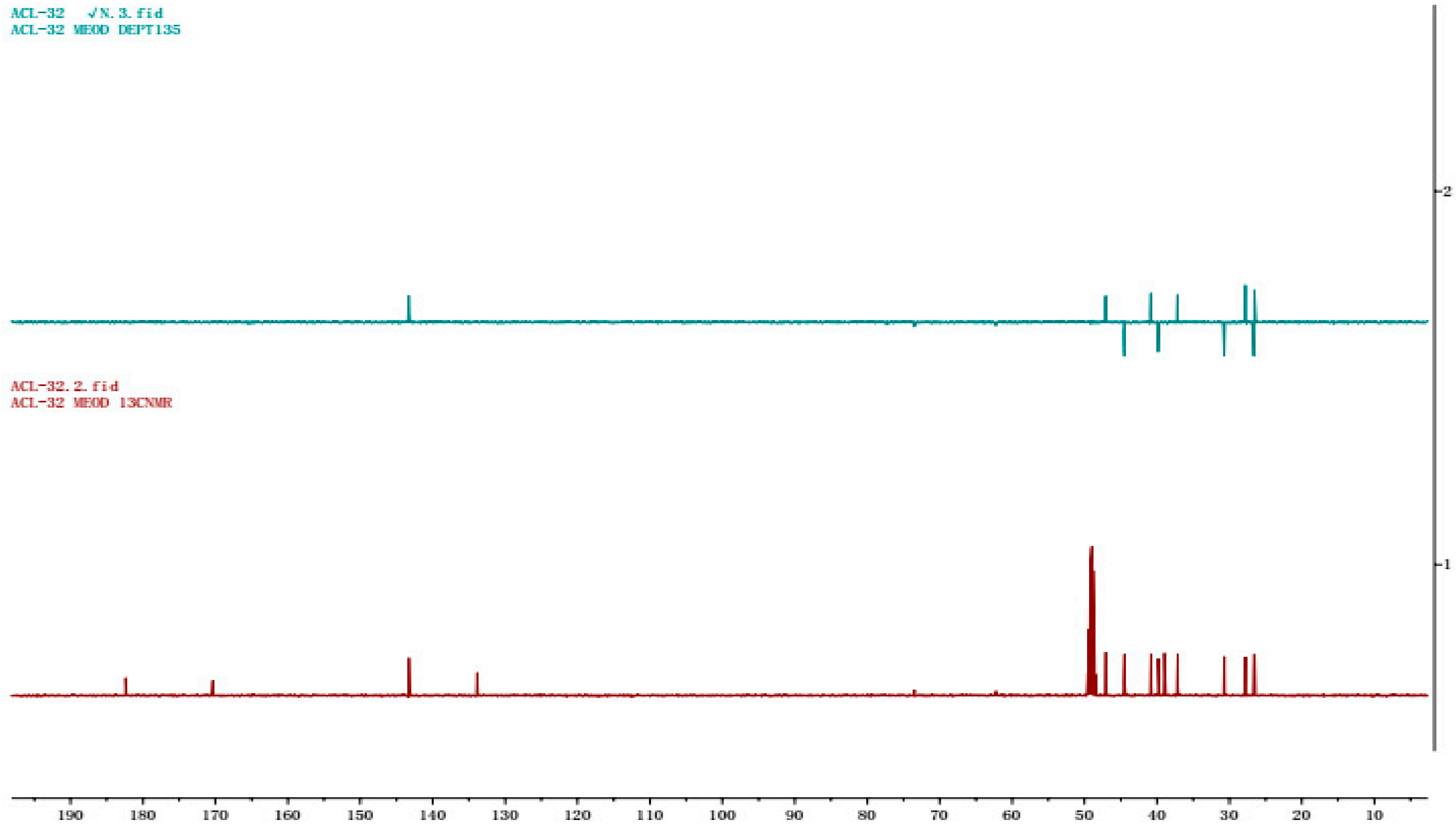

Figure S4. The HSQC spectrum of compound **1** in CD_3OD

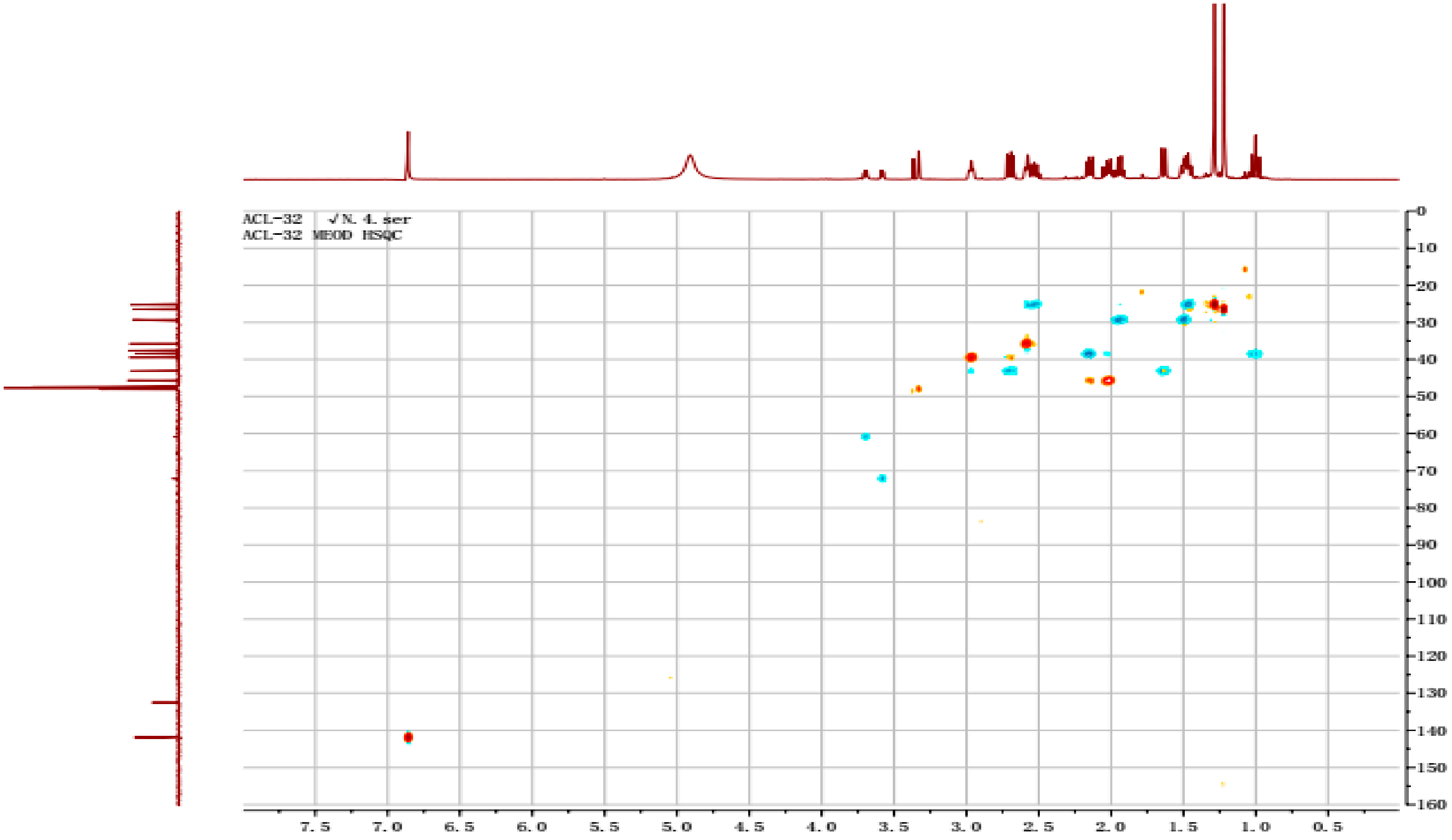

Figure S5. The ^{1}H-^{1}H COSY spectrum of compound **1** in CD_3OD

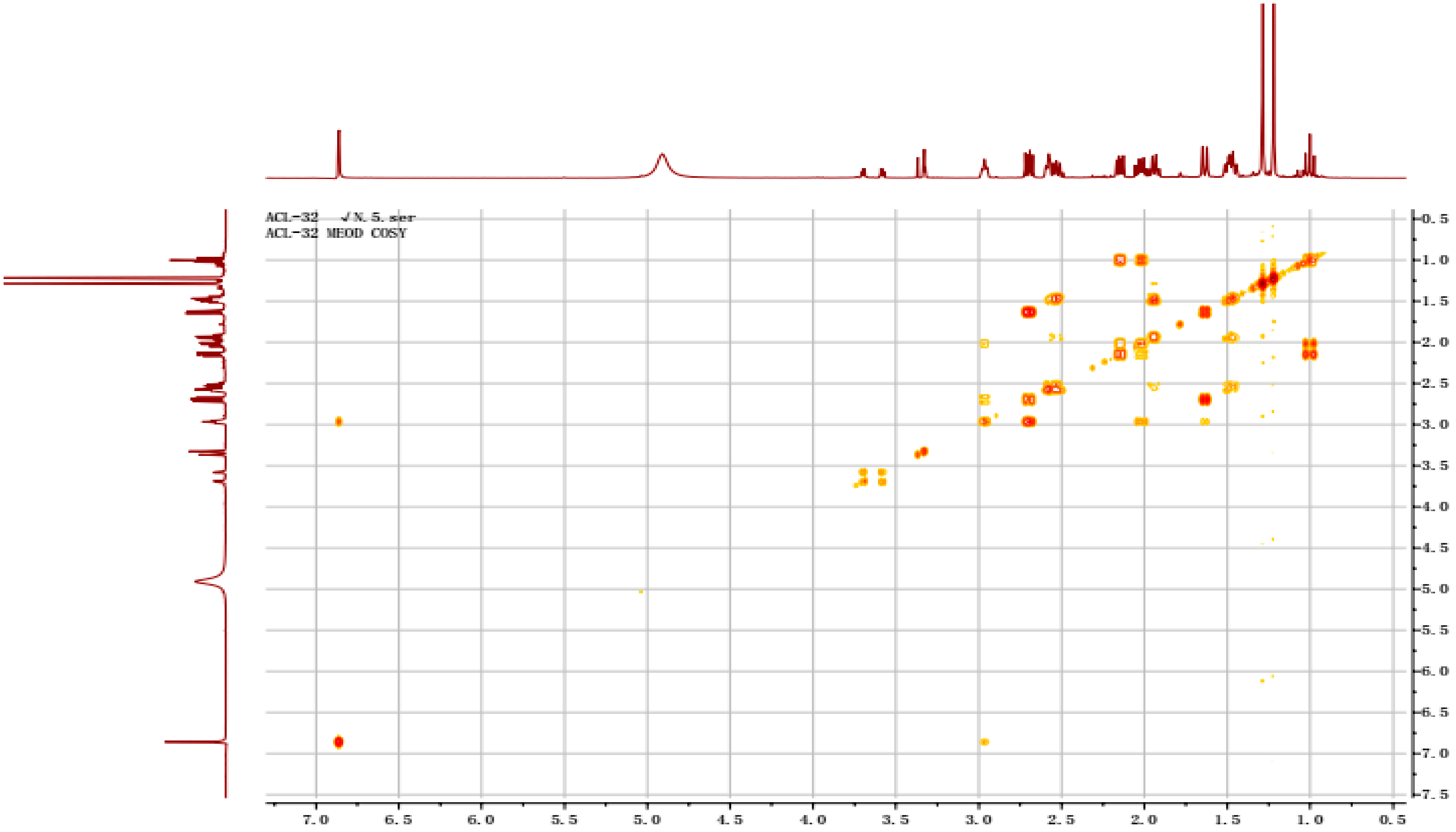

Figure S6. The HMBC spectrum of compound **1** in CD_3OD

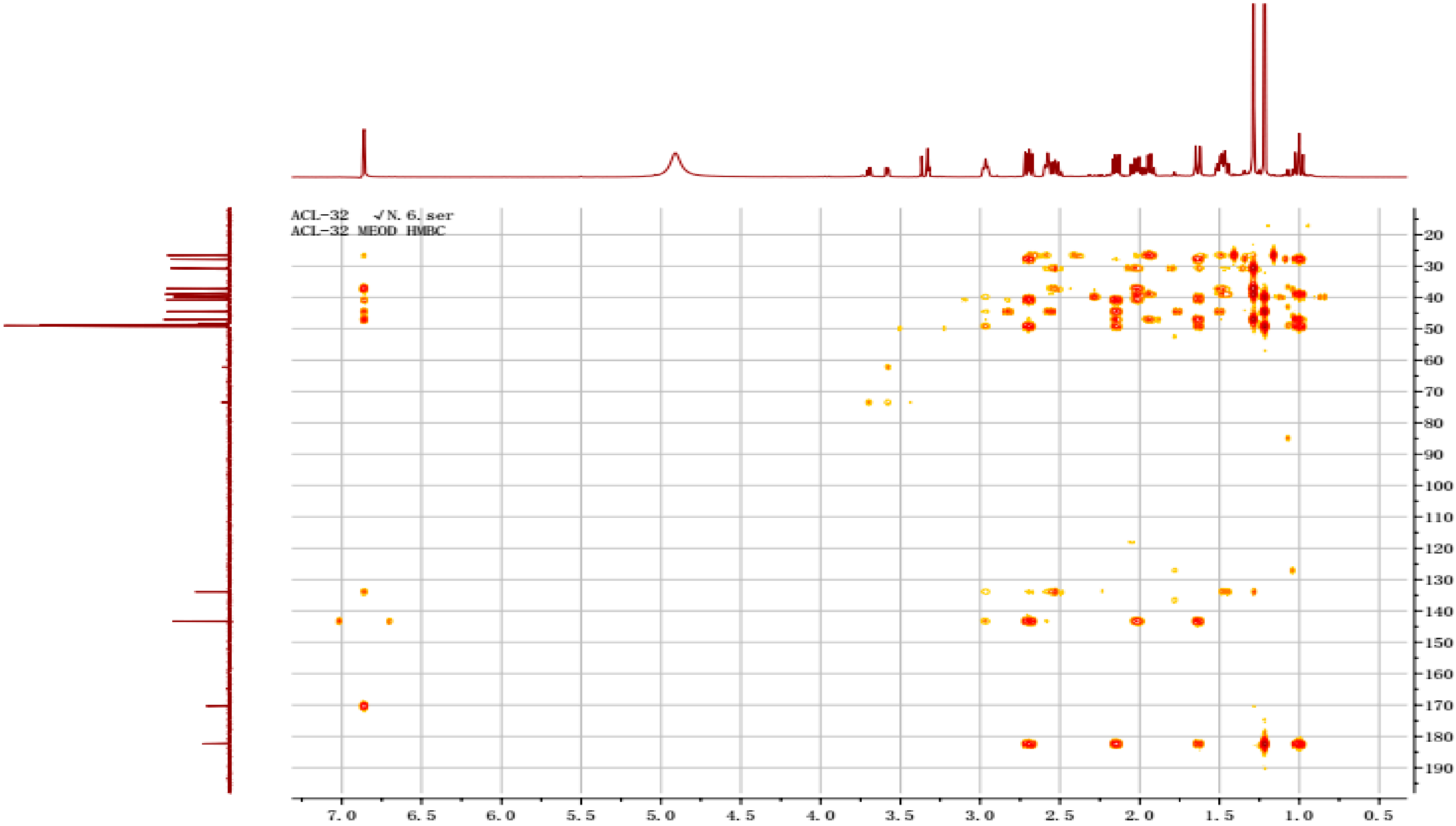

Figure S7. The ROESY spectrum of compound **1** in CD_3OD

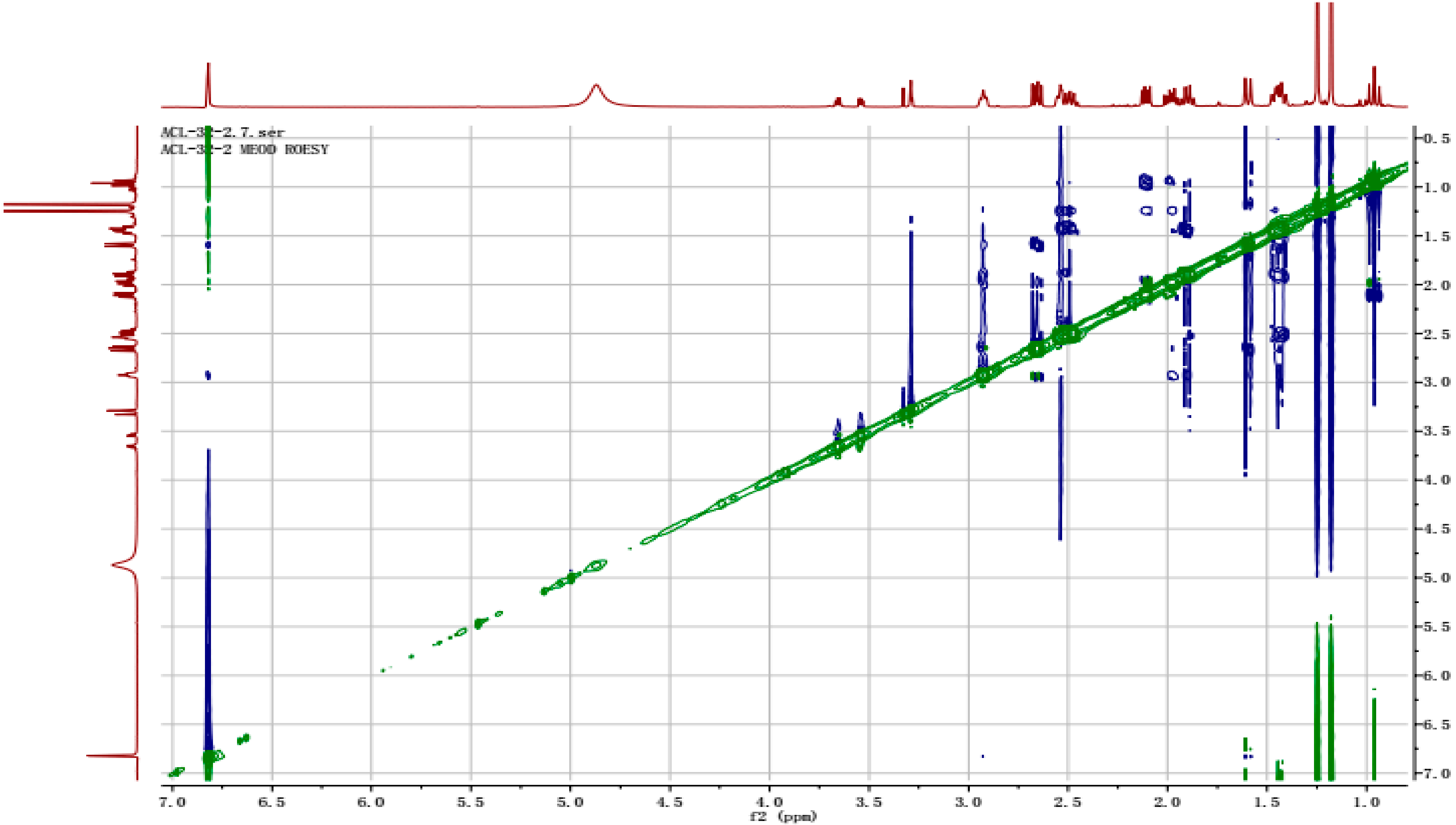

Figure S8. The HRESIMS and IR spectra of compound 1

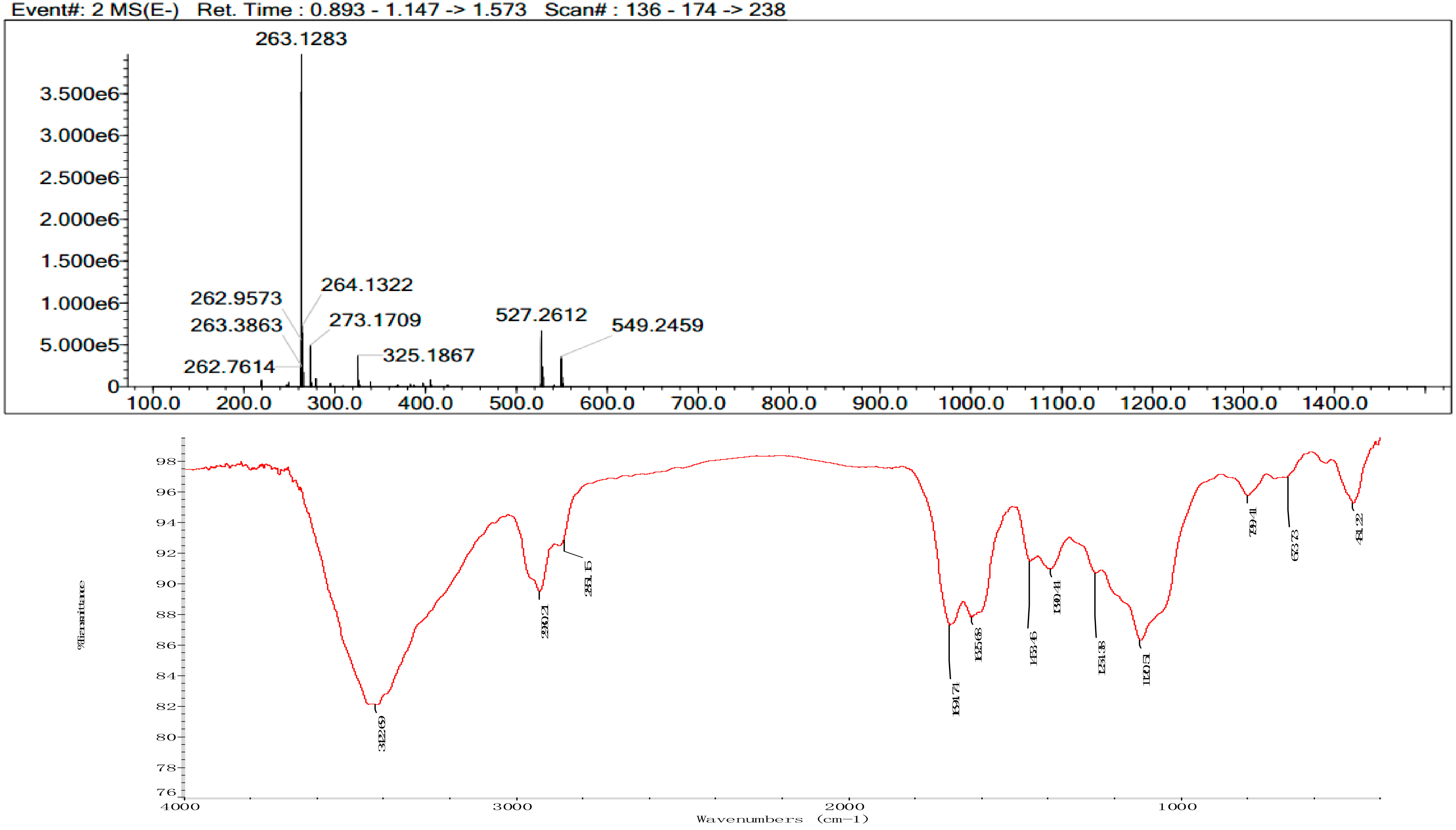

Figure S9. The ^{1}H NMR spectrum of compound **2** in $CDCl_3$

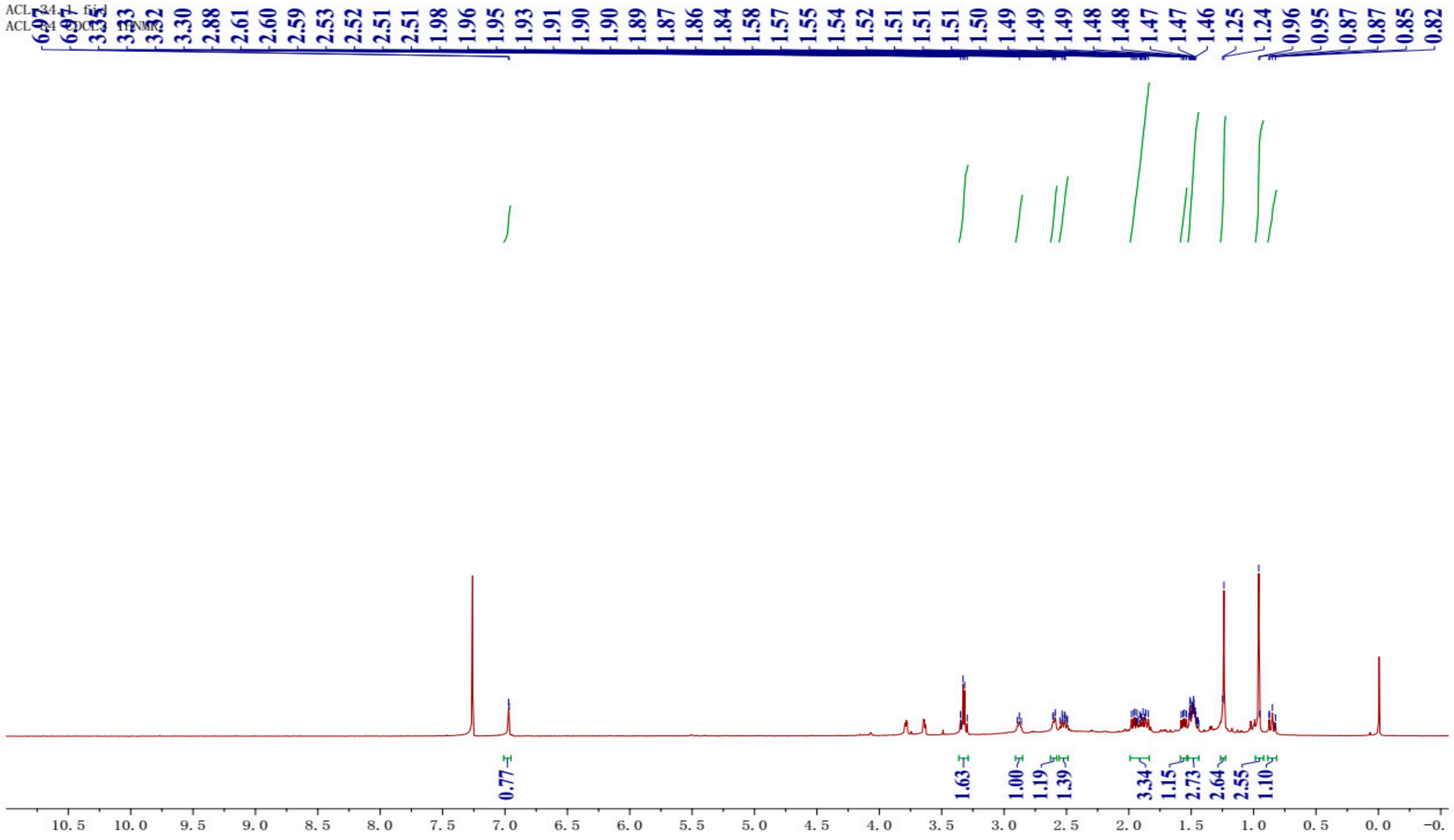

Figure S10. The ^{13}C-NMR spectrum of compound **2** in $CDCl_3$

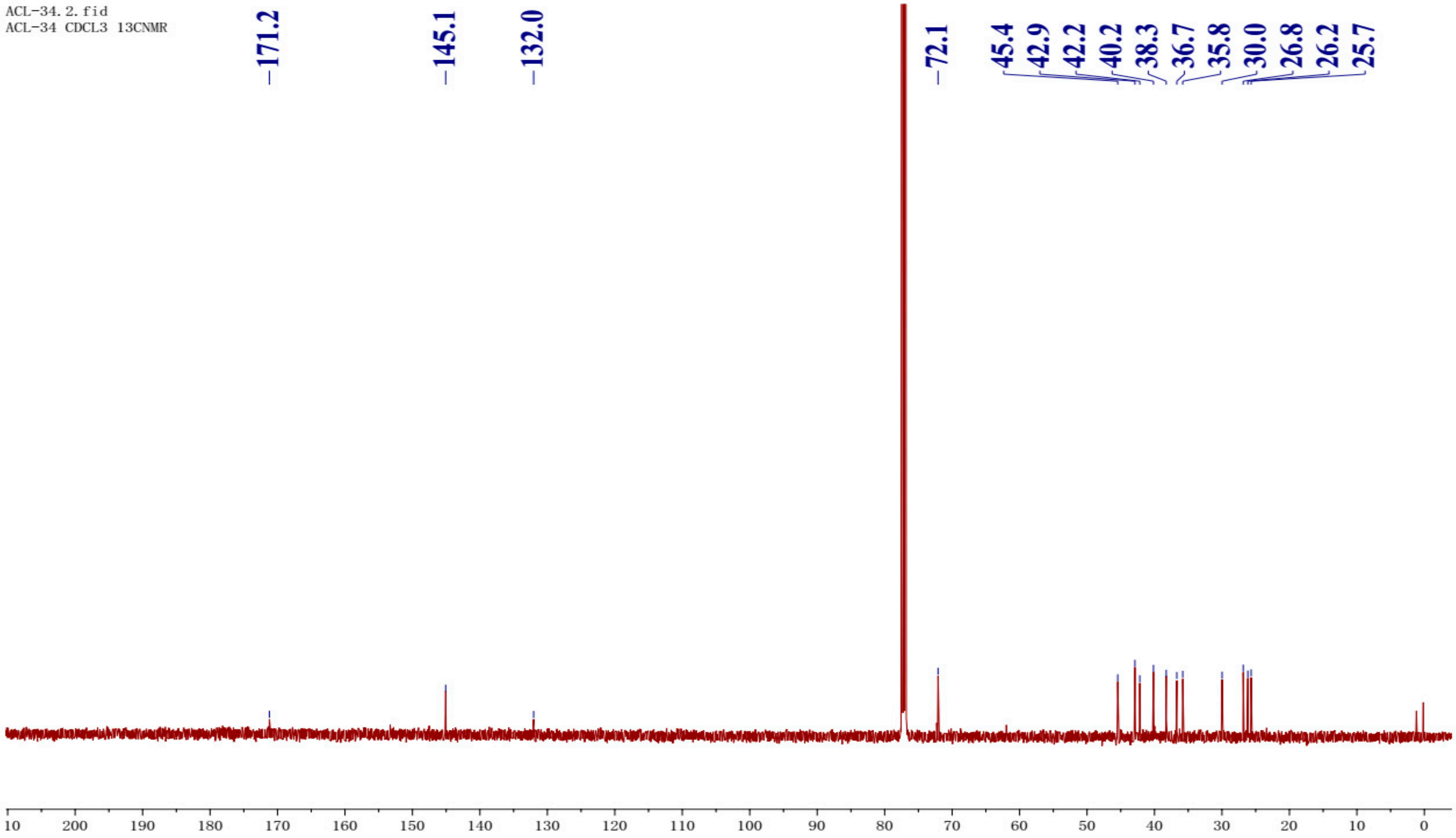

Figure S11. The DEPT spectrum of compound **2** in $CDCl_3$

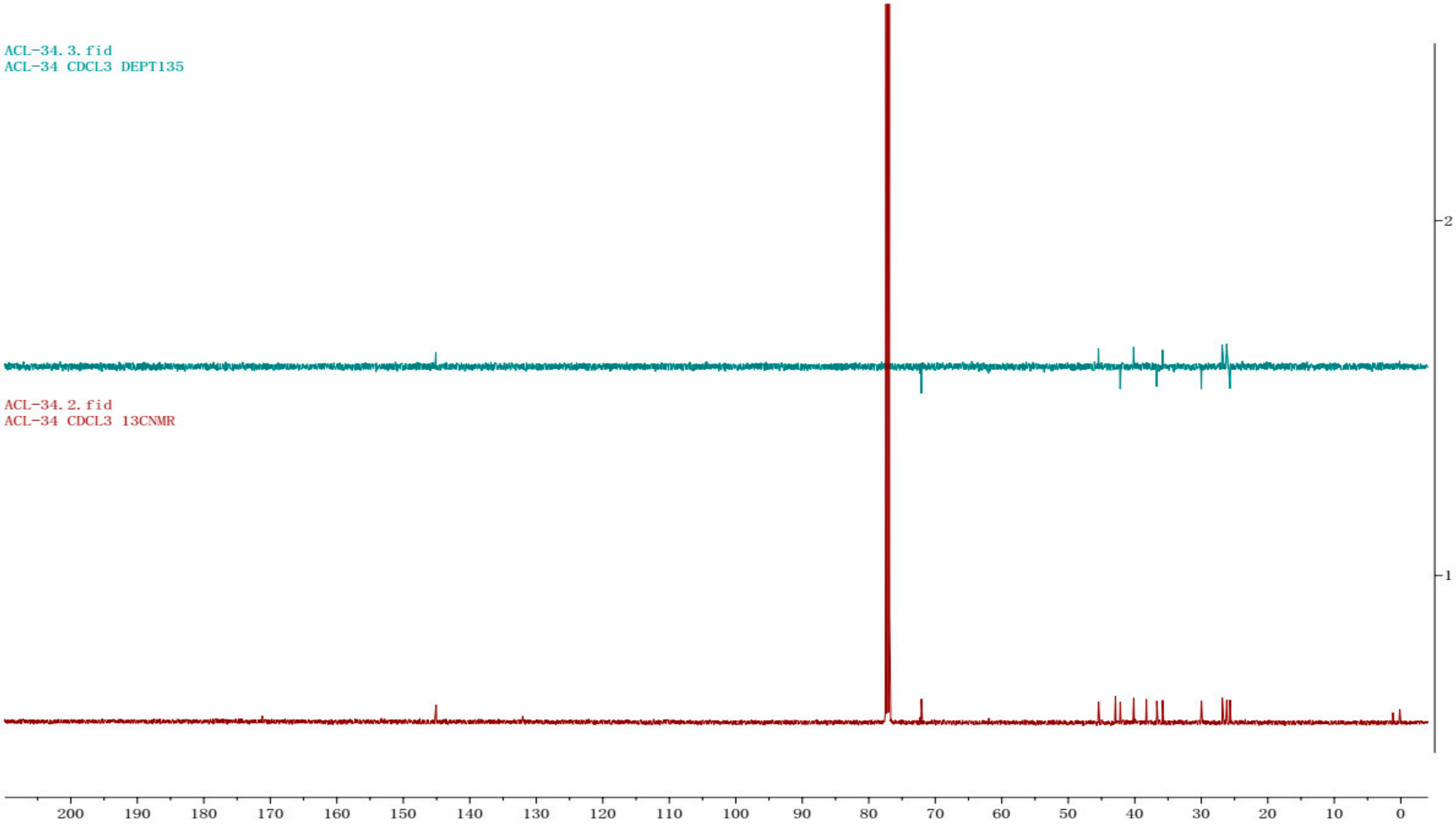

Figure S12. The HSQC spectrum of compound **2** in $CDCl_3$

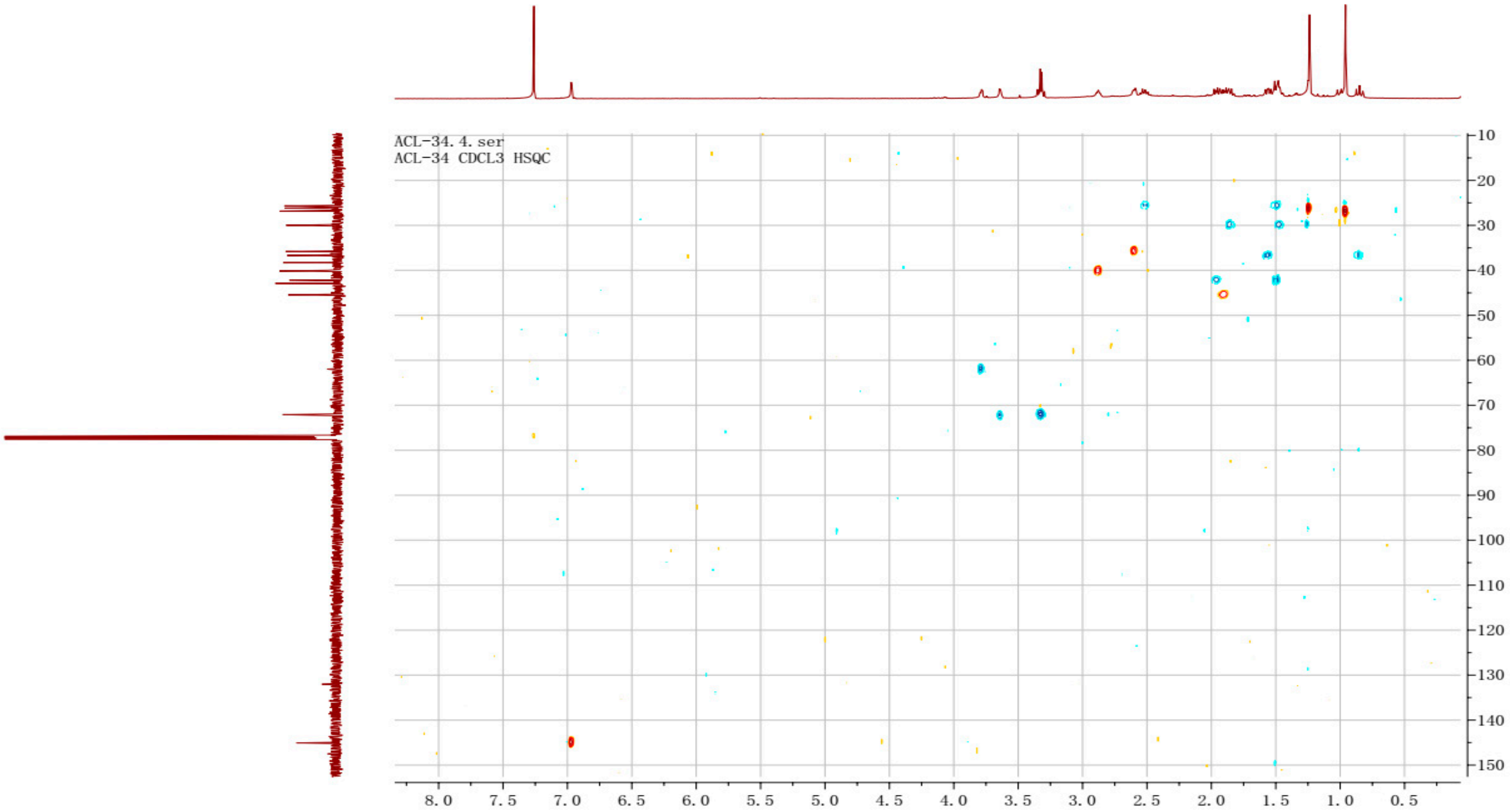

Figure S13. The ^{1}H-^{1}H COSY spectrum of compound **2** in $CDCl_3$

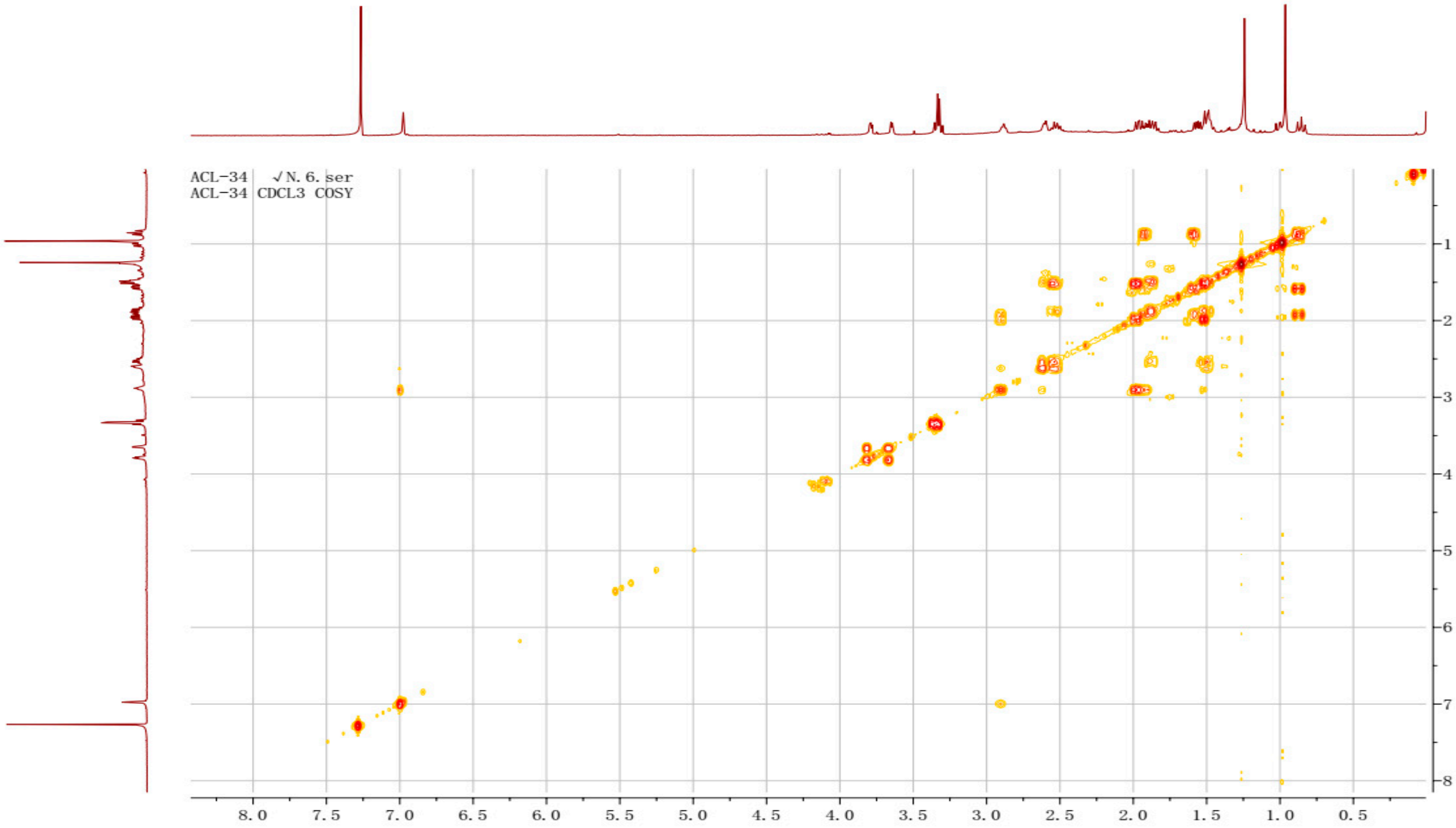

Figure S14. The HMBC spectrum of compound **2** in $CDCl_3$

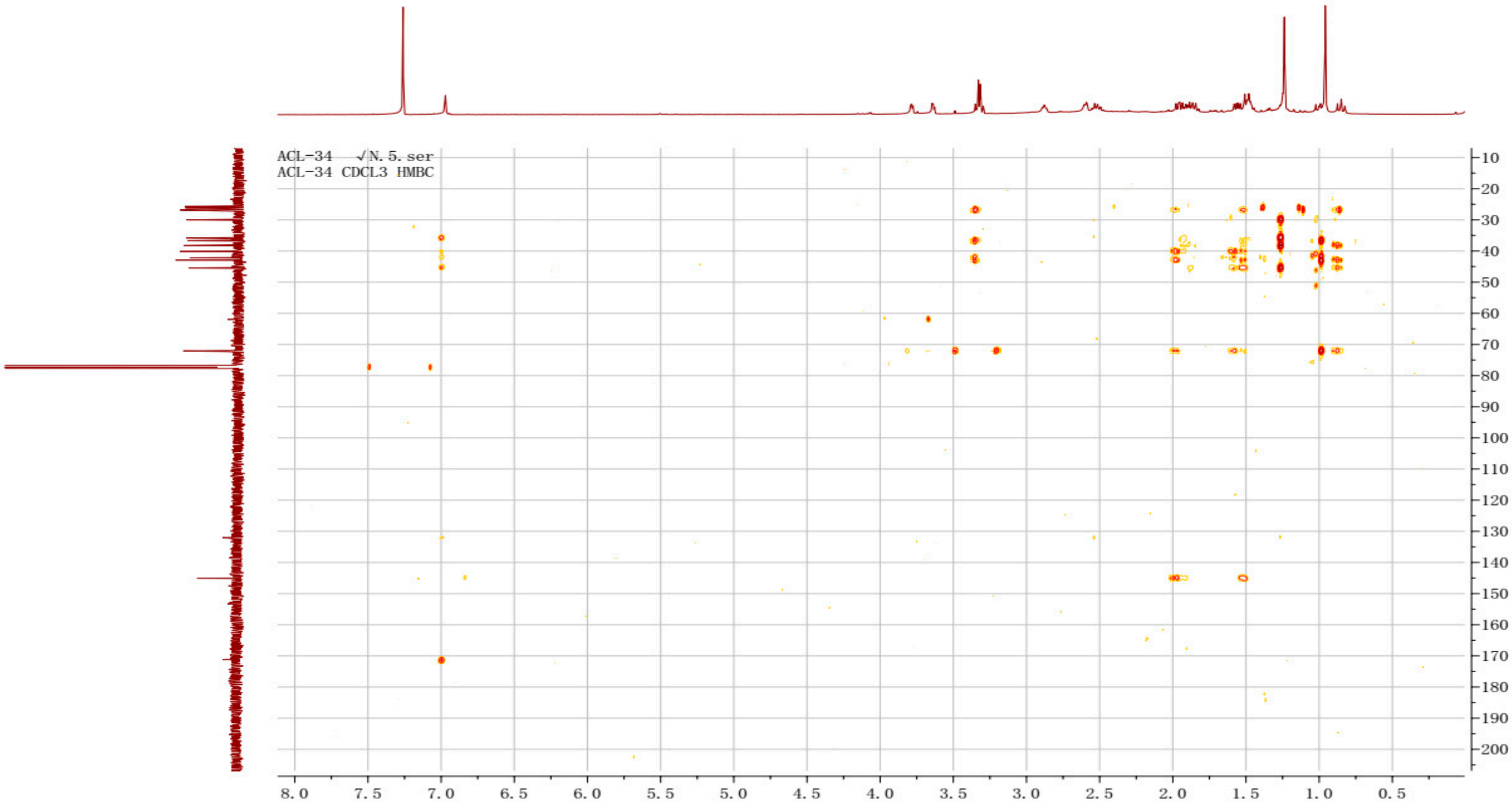

Figure S15. The ROESY spectrum of compound **2** in $CDCl_3$

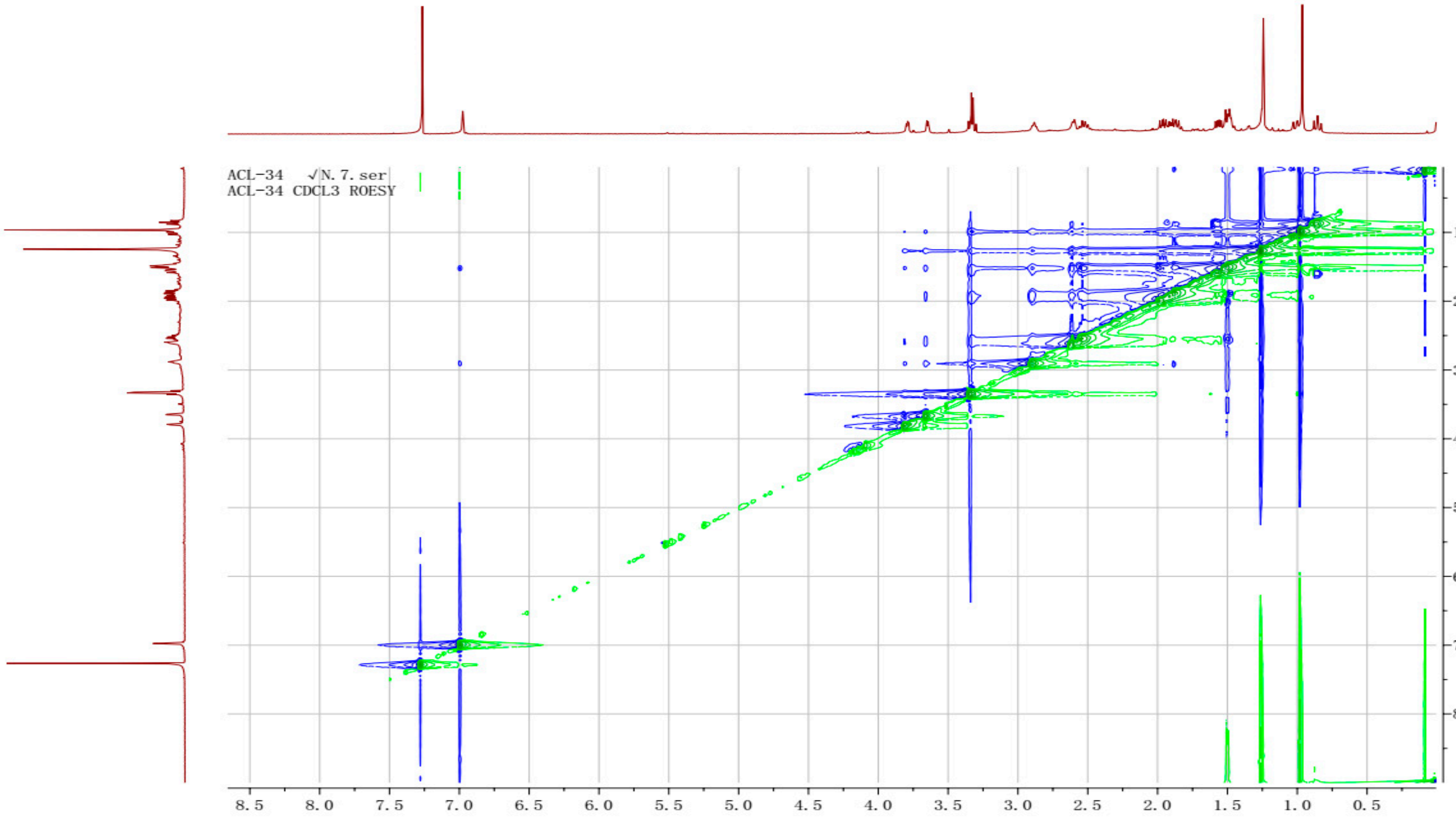

Figure S16. The HRESIMS and IR spectra of compound **2**

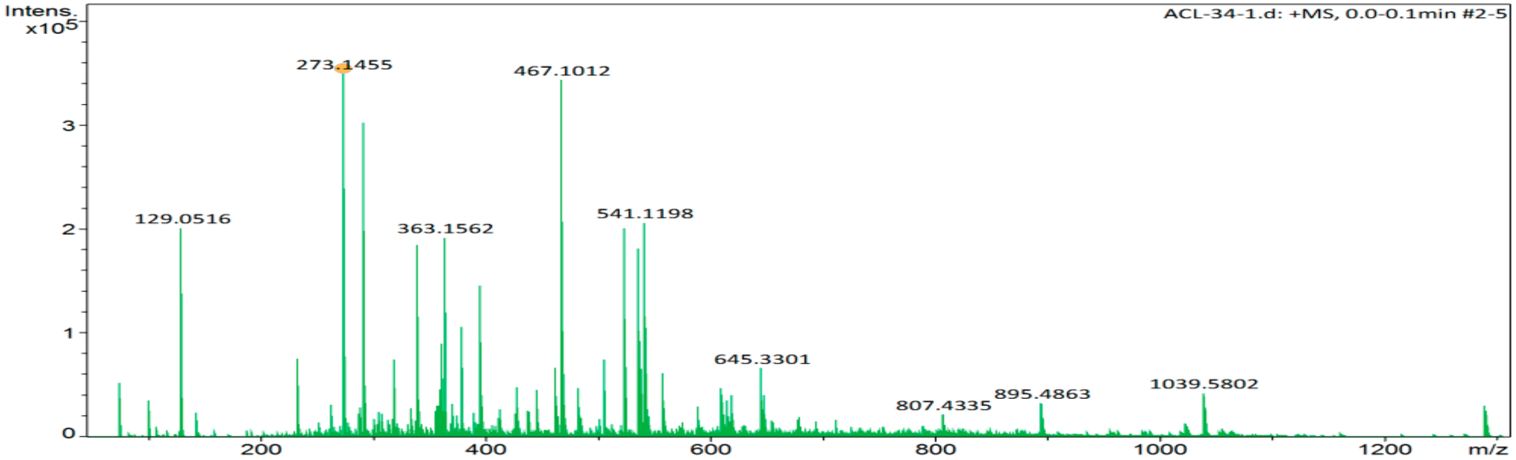

Sum Formula	Ion Formula	Meas. m/z	m/z	err [mDa]	err [ppm]
C15H22O3	C15H22NaO3	273.1455	273.1461	0.6	2.1

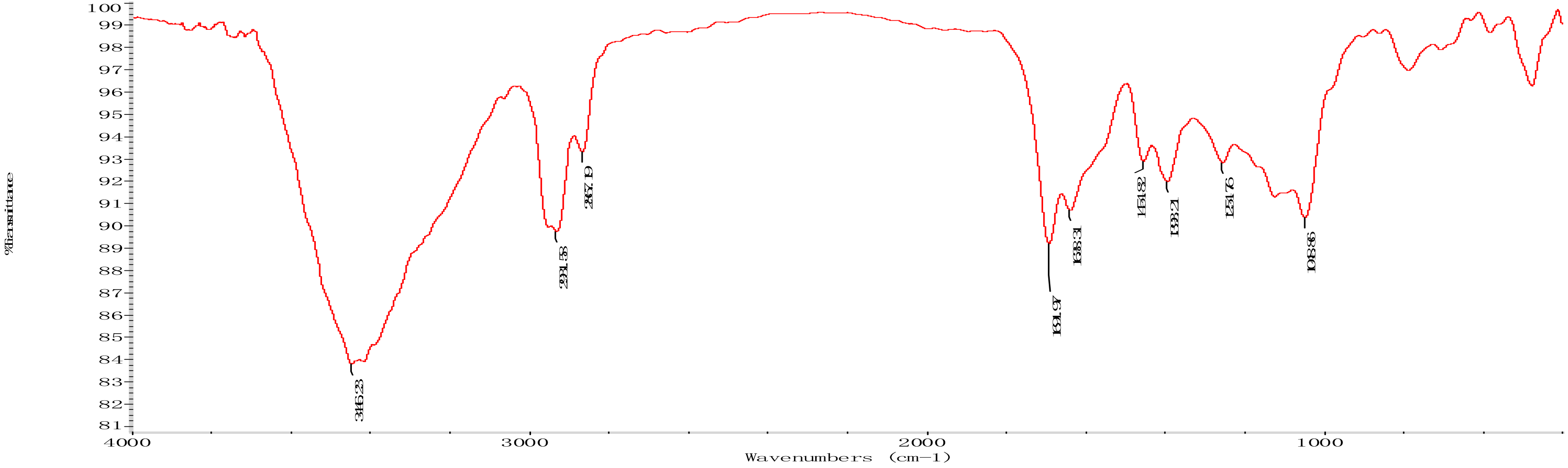

Figure S17. The ^{1}H NMR spectrum of compound **3** in $CDCl_3$

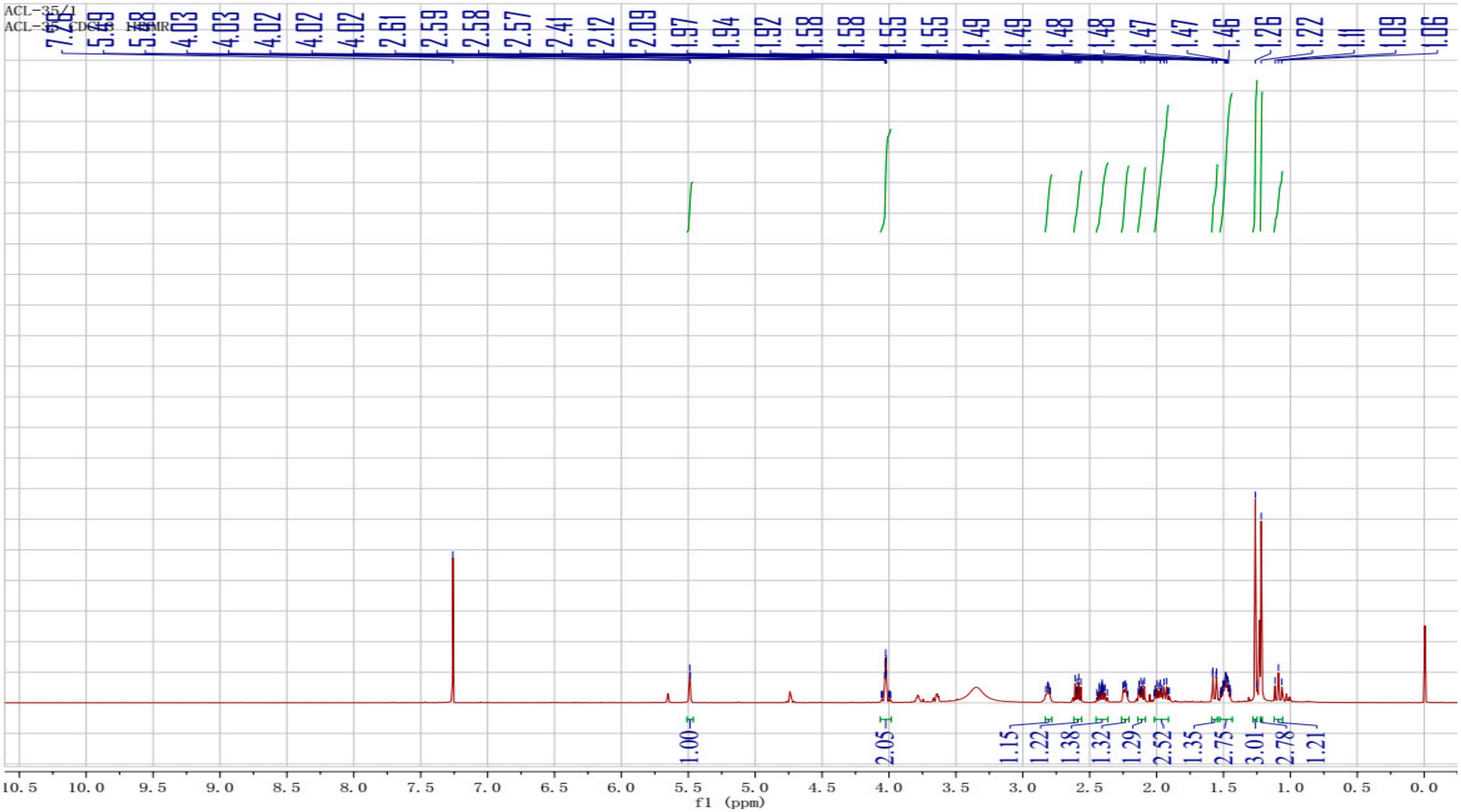

Figure S18. The ^{13}C-NMR spectrum of compound **3** in $CDCl_3$

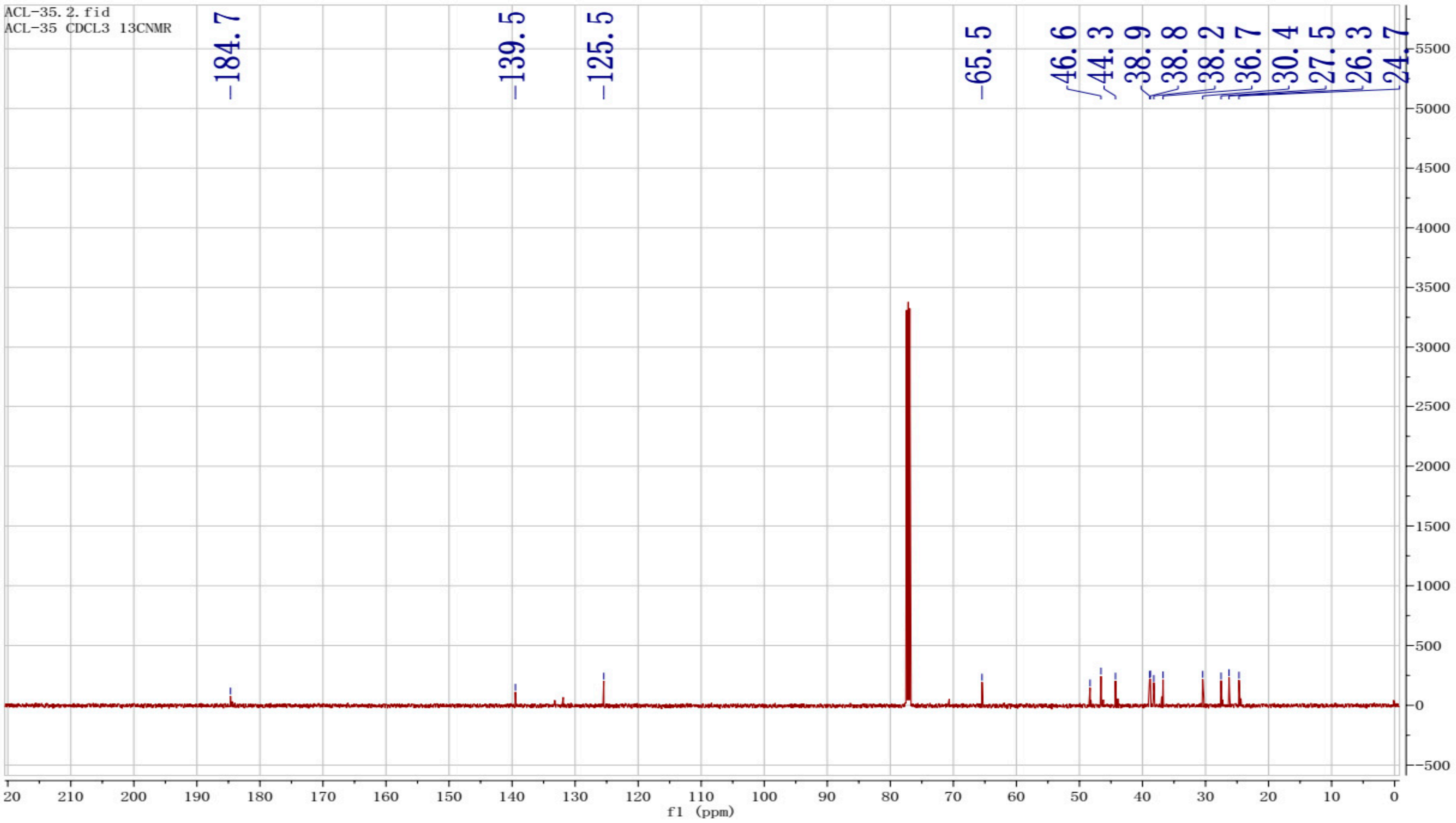

Figure S19. The DEPT spectrum of compound **3** in $CDCl_3$

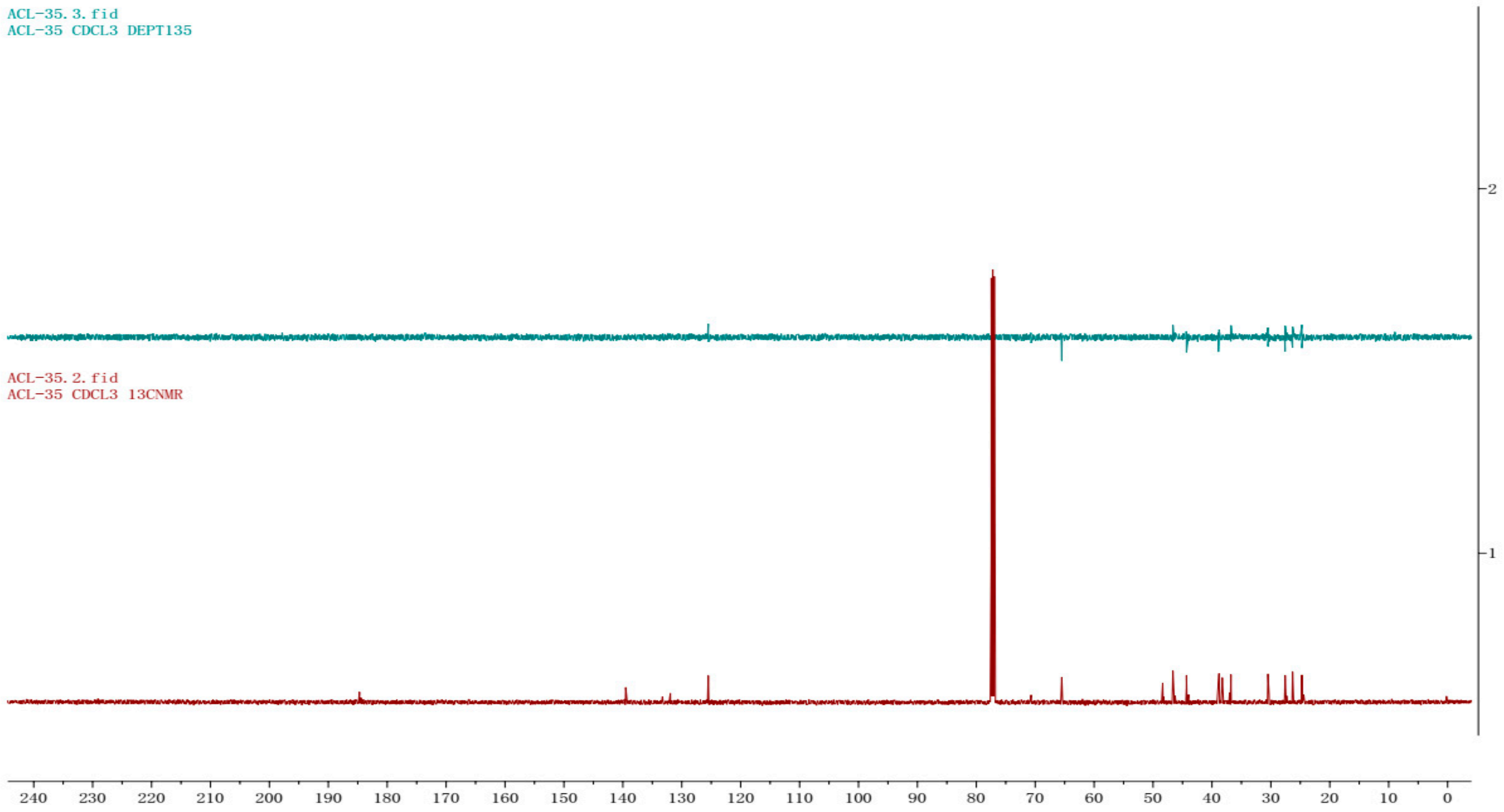

Figure S20. The HSQC spectrum of compound **3** in $CDCl_3$

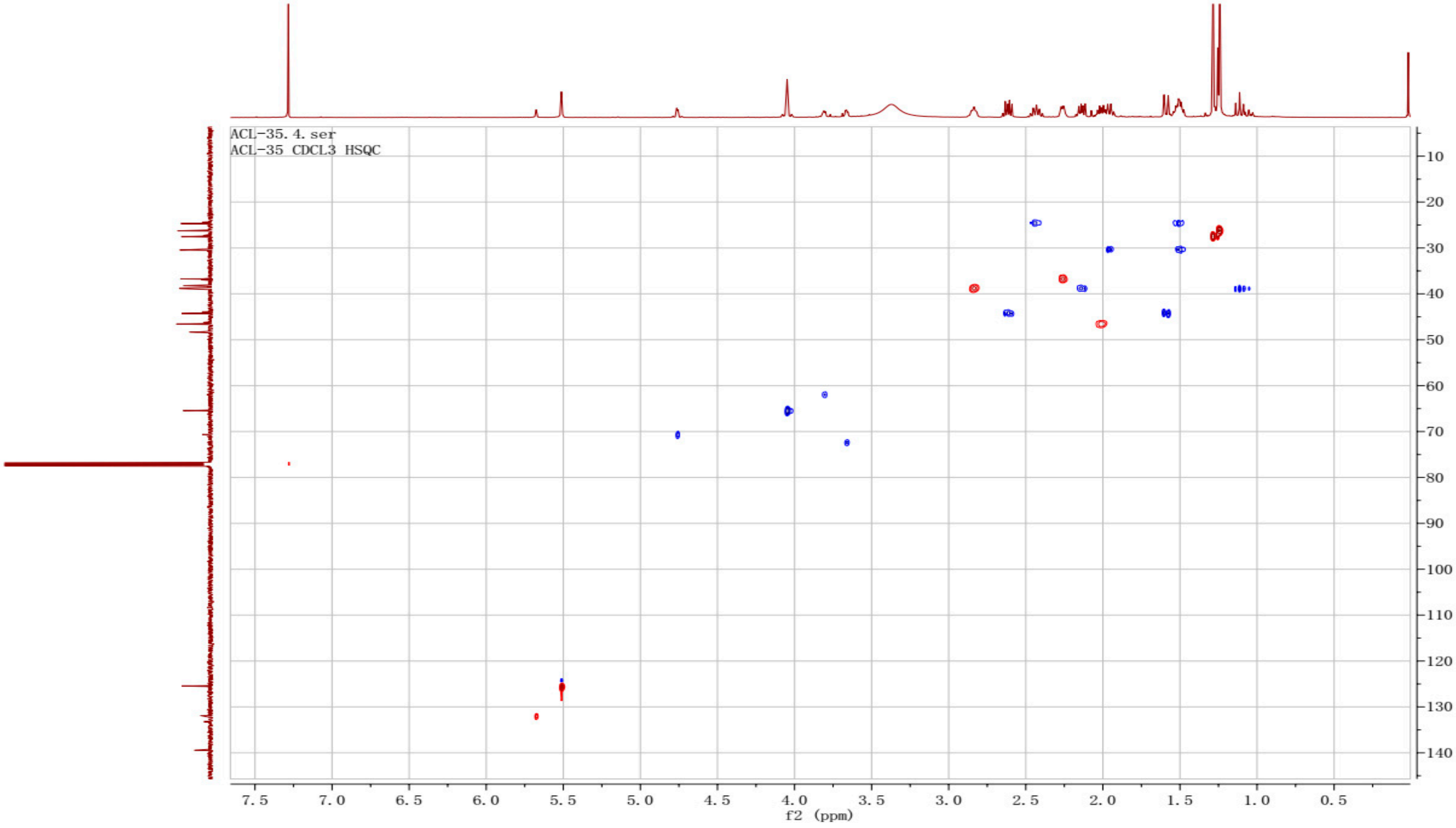

Figure S21. The ^{1}H-^{1}H COSY spectrum of compound **3** in CDCl3

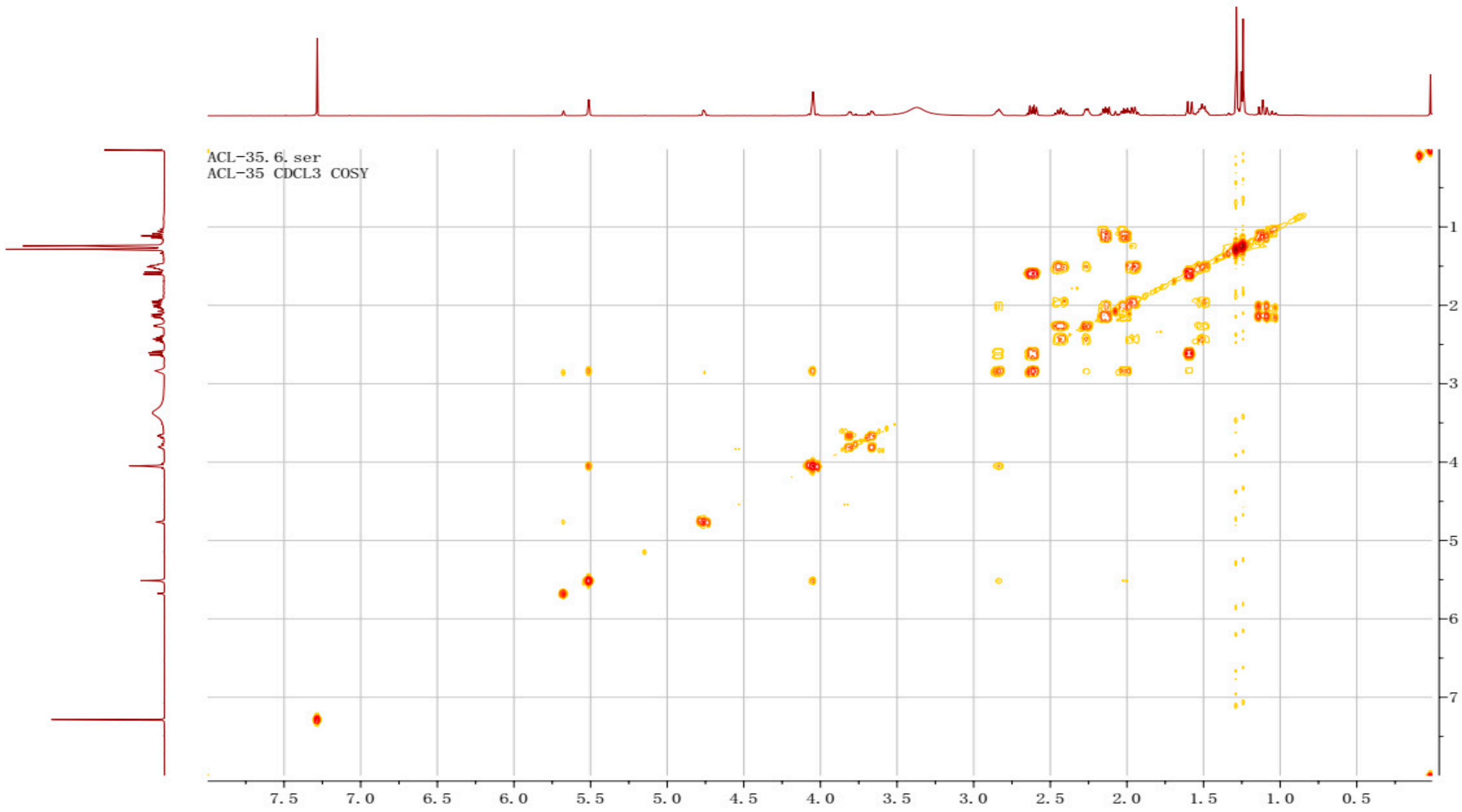

Figure S22. The HMBC spectrum of compound **3** in $CDCl_3$

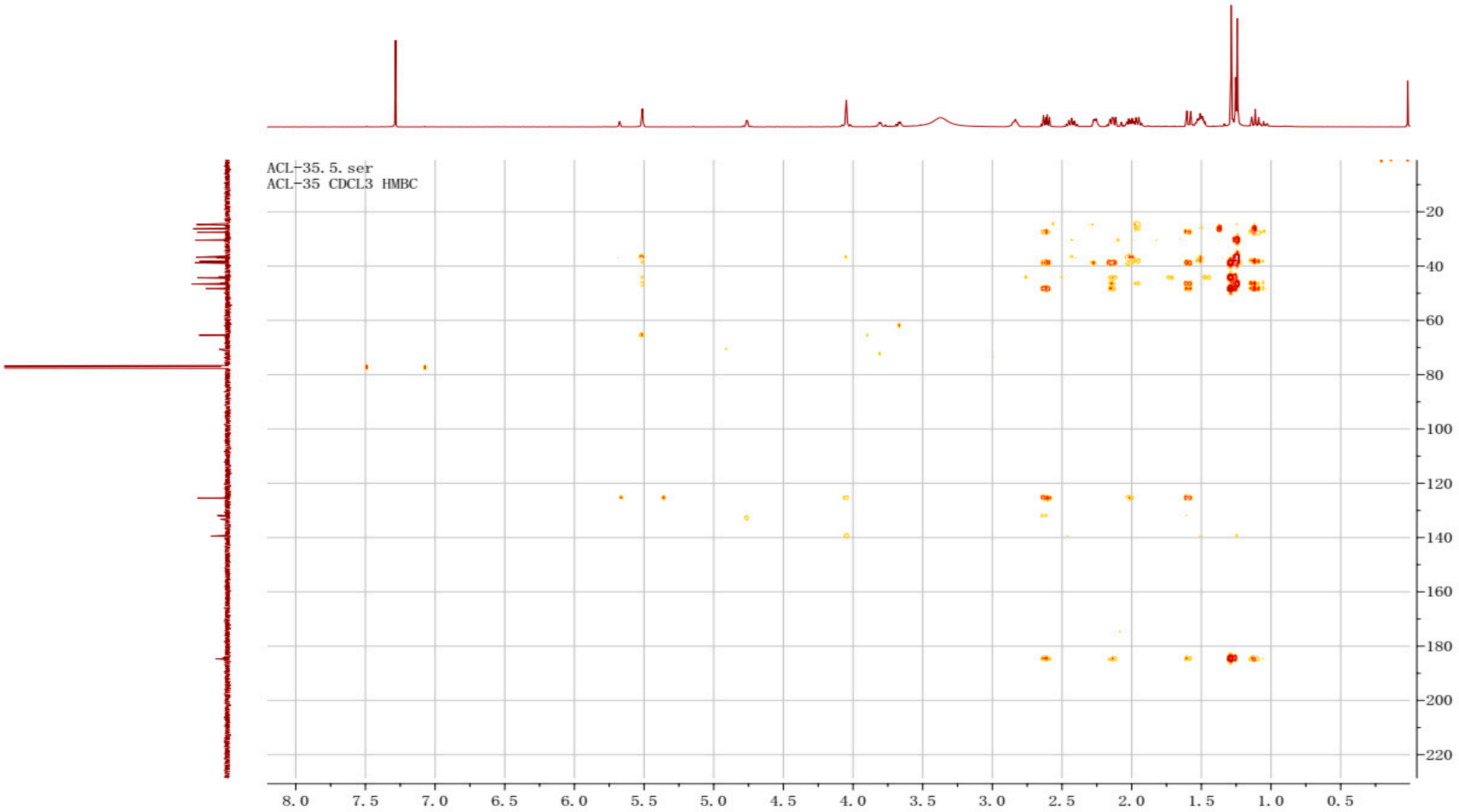

Figure S23. The ROESY spectrum of compound **3** in $CDCl_3$

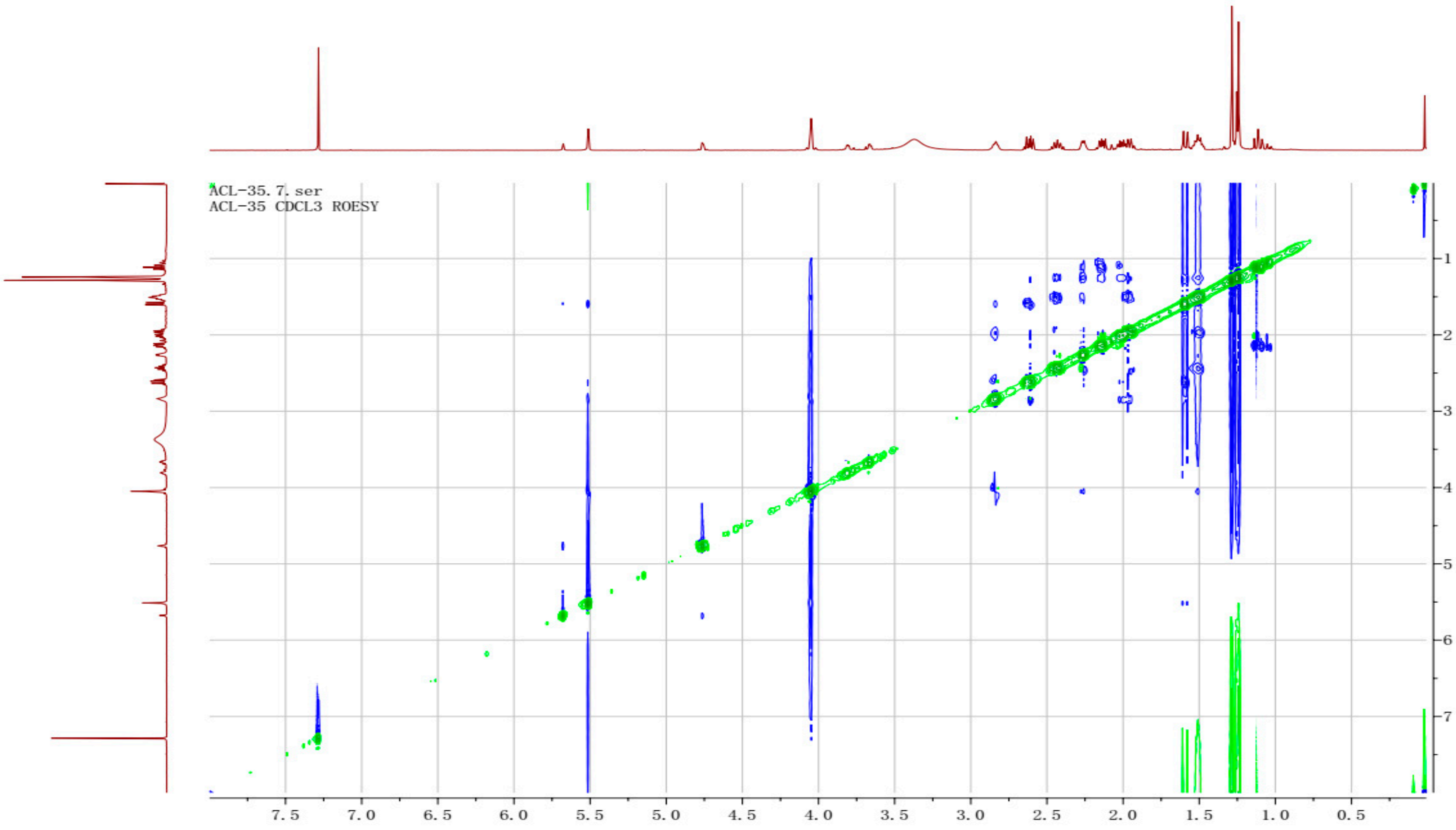

Figure S24. The HRESIMS and IR spectra of compound **3**

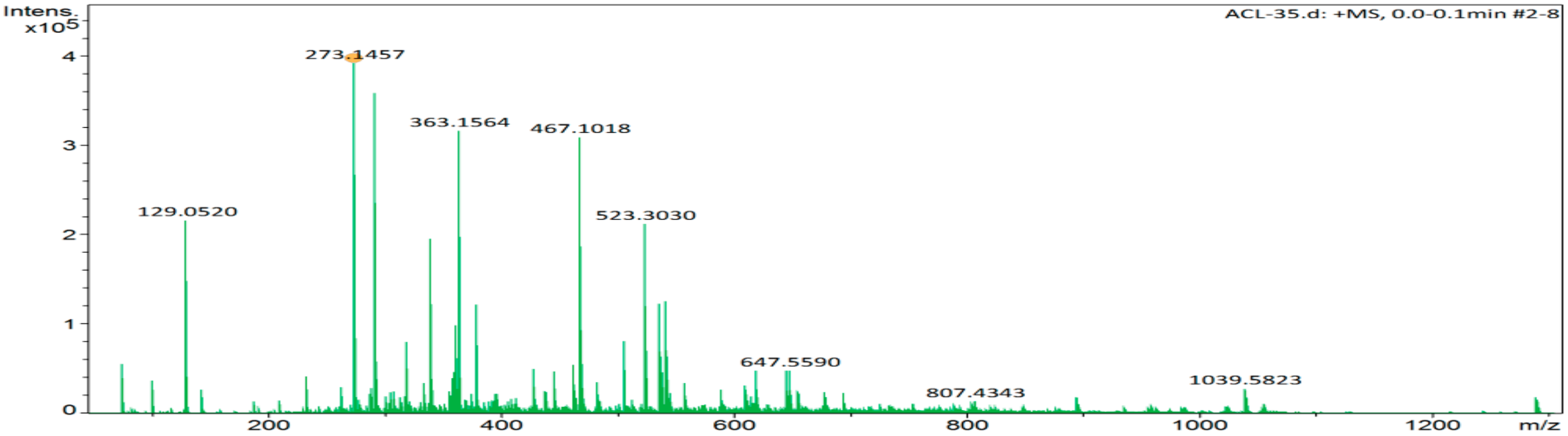

Sum Formula	Ion Formula	Meas. m/z	m/z	err [mDa]	err [ppm]
C15H22O3	C15H22NaO3	273.1457	273.1461	0.4	1.4

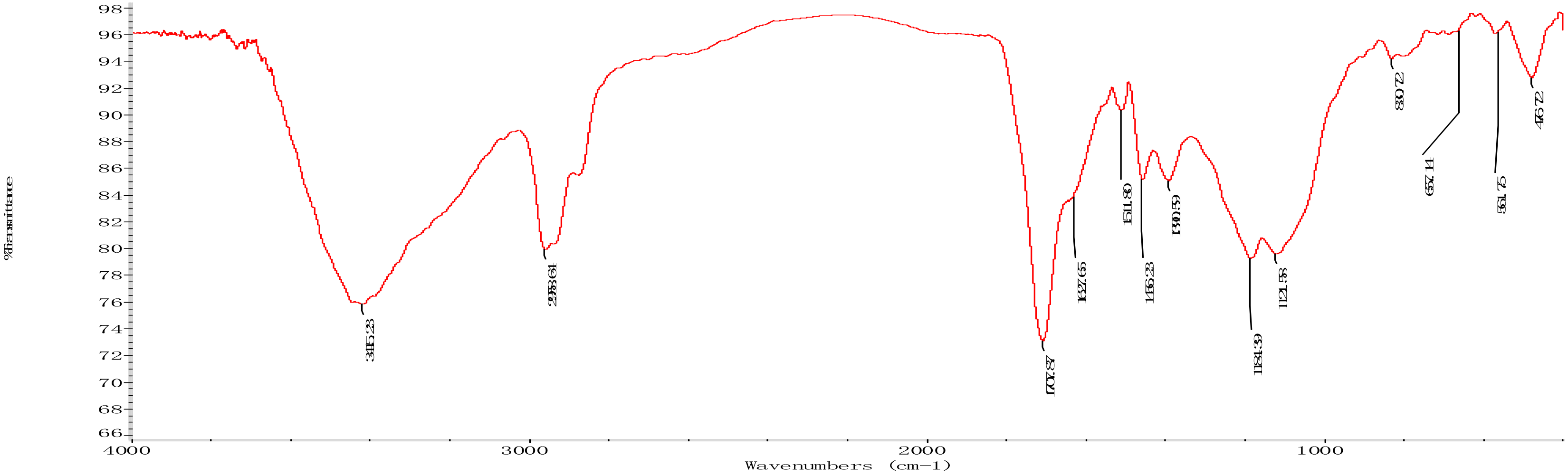

Figure S25. The ^{1}H NMR spectrum of compound **4** in $CDCl_3$

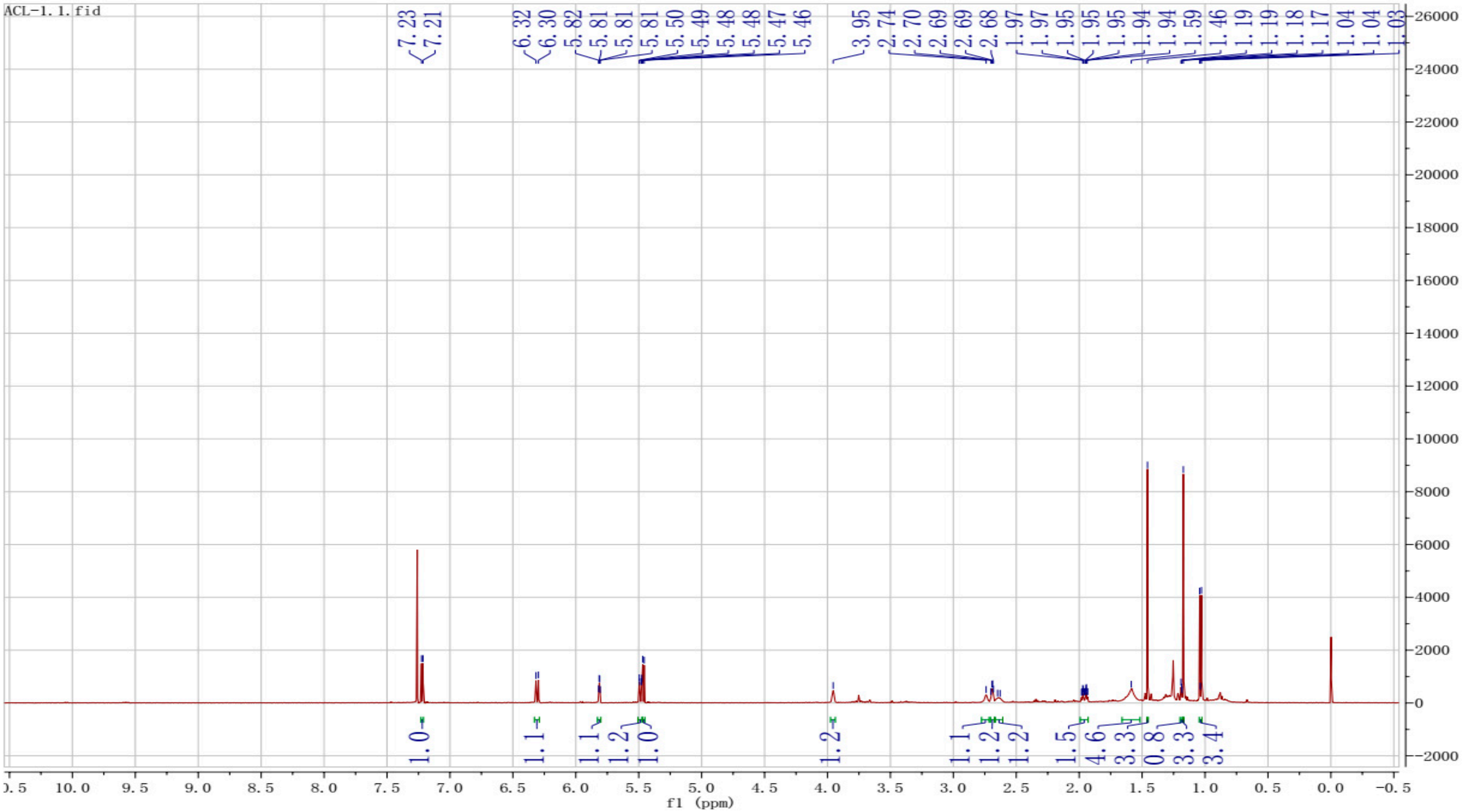

Figure S26. The ^{13}C-NMR spectrum of compound **4** in $CDCl_3$

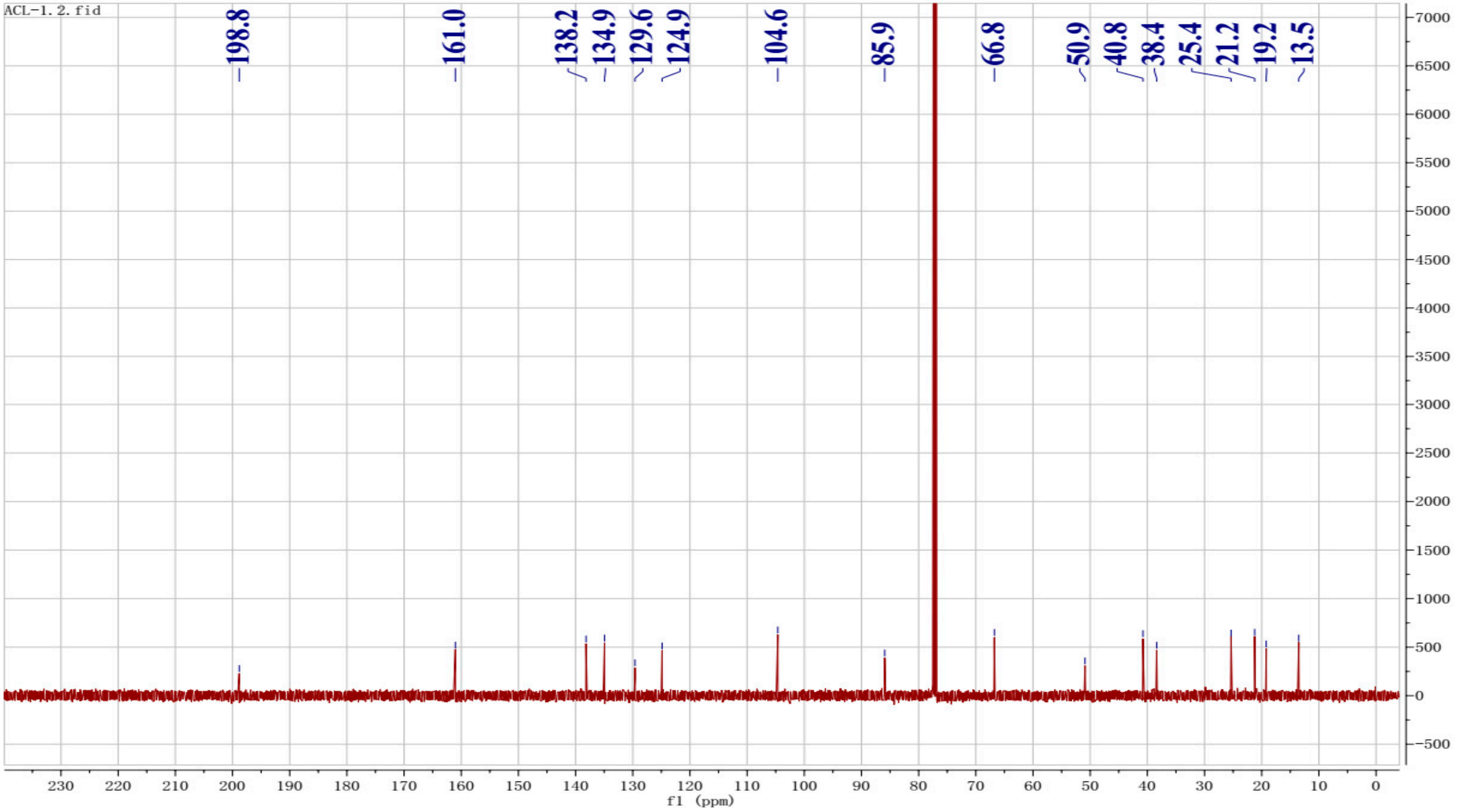

Figure S27. The DEPT spectrum of compound **4** in $CDCl_3$

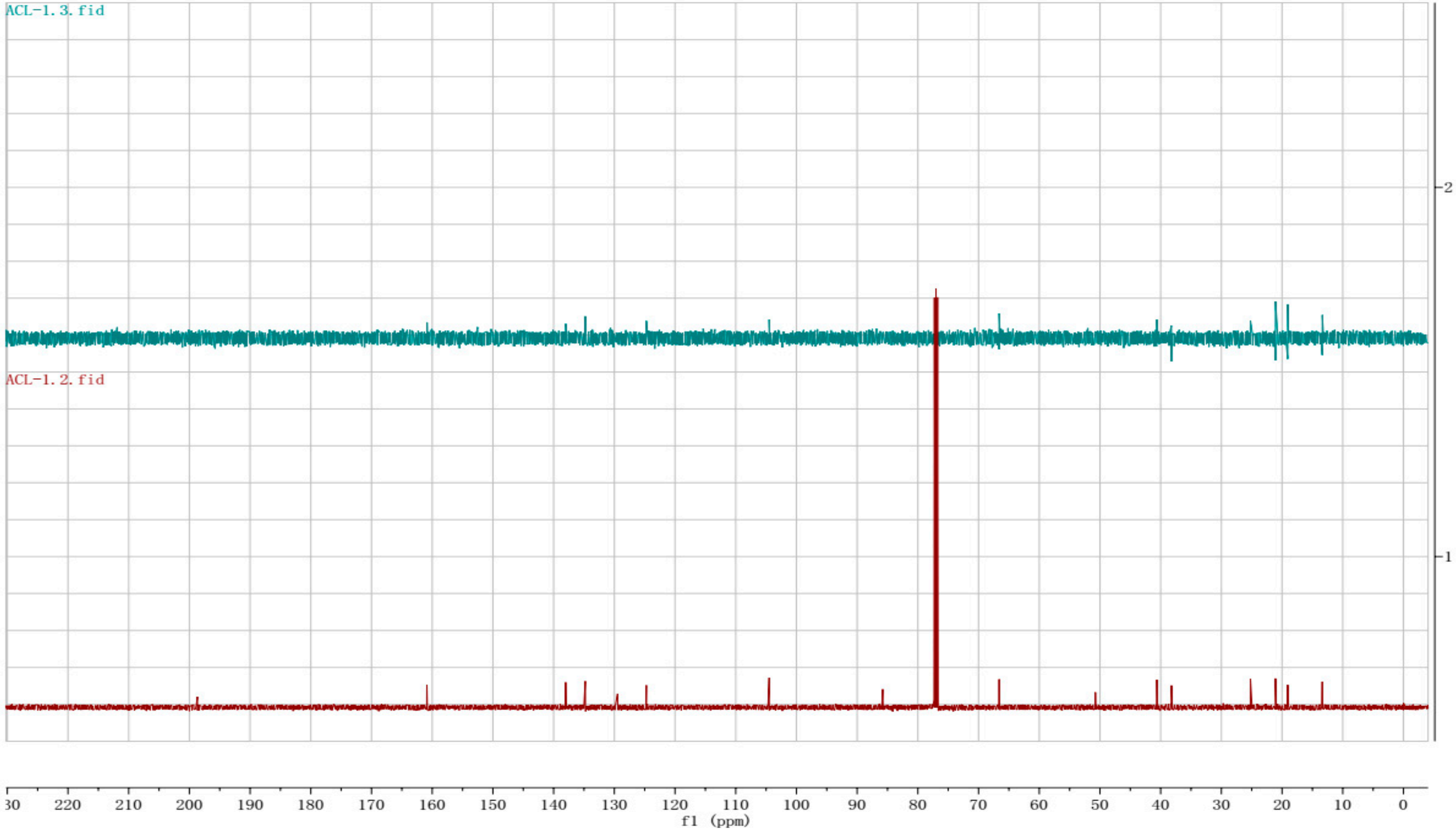

Figure S28. The HSQC spectrum of compound **4** in $CDCl_3$

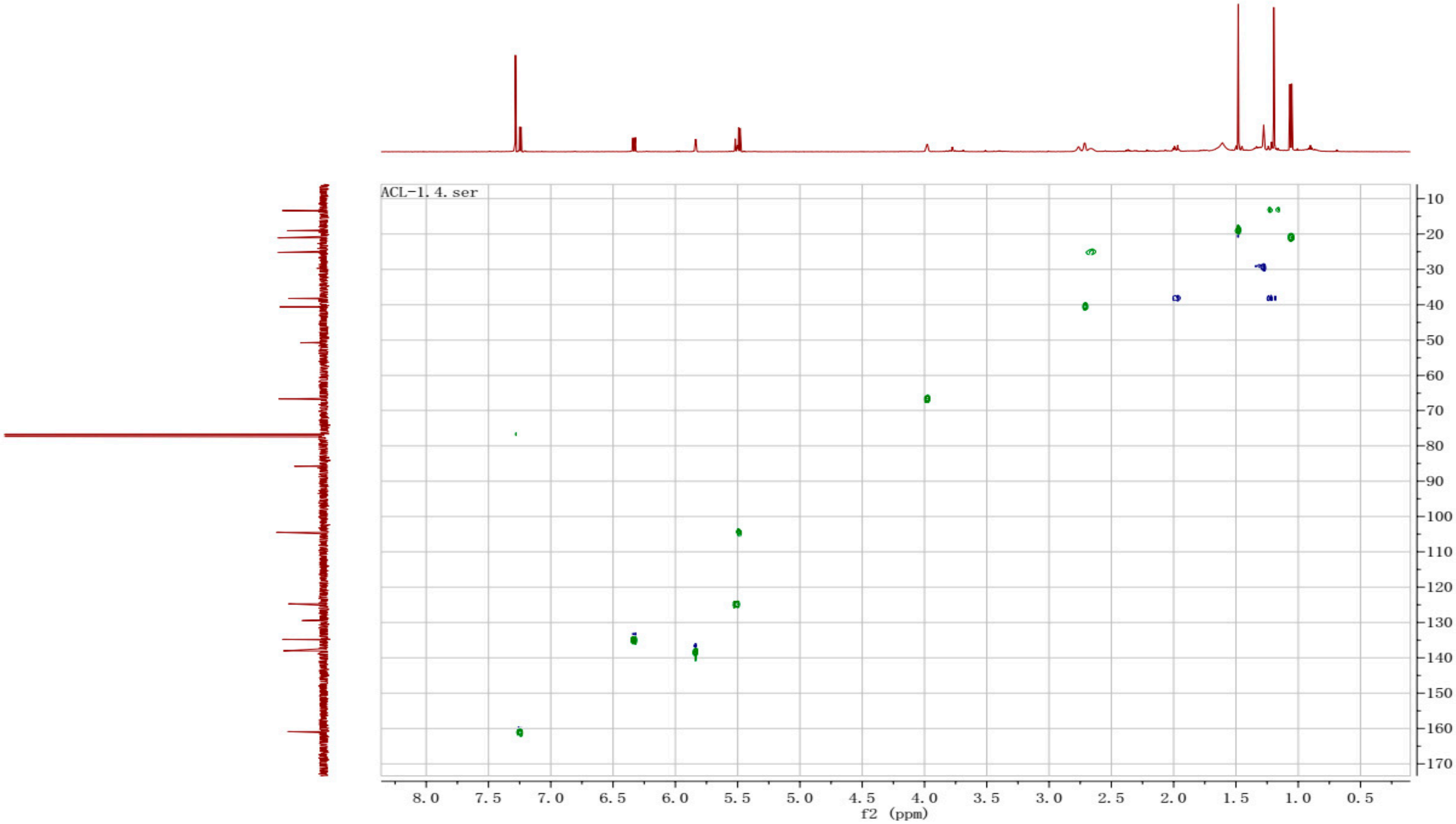

Figure S29. The ^1H-^1H COSY spectrum of compound **4** in $CDCl_3$

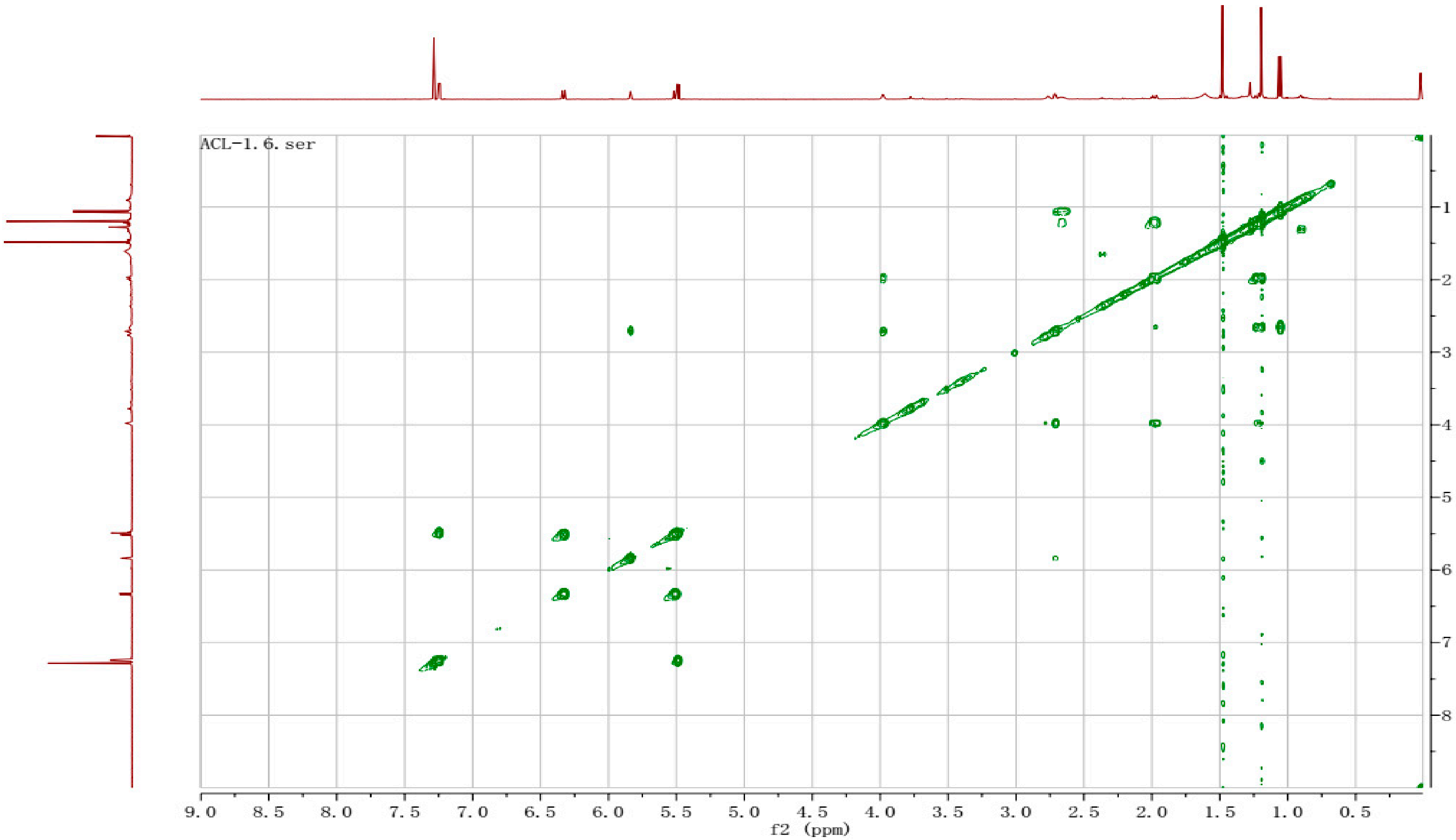

Figure S30. The HMBC spectrum of compound **4** in $CDCl_3$

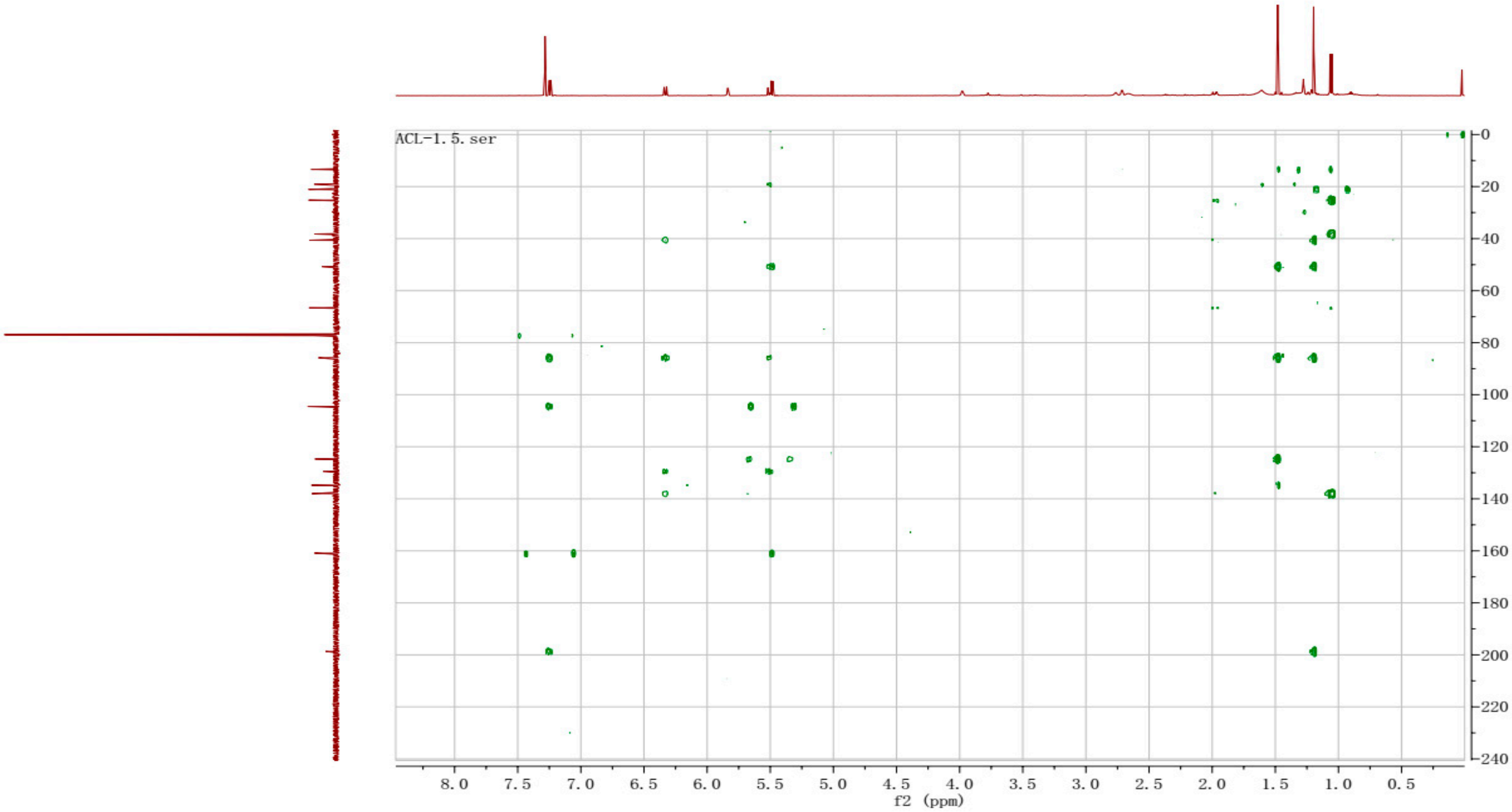

Figure S31. The ROESY spectrum of compound **4** in $CDCl_3$

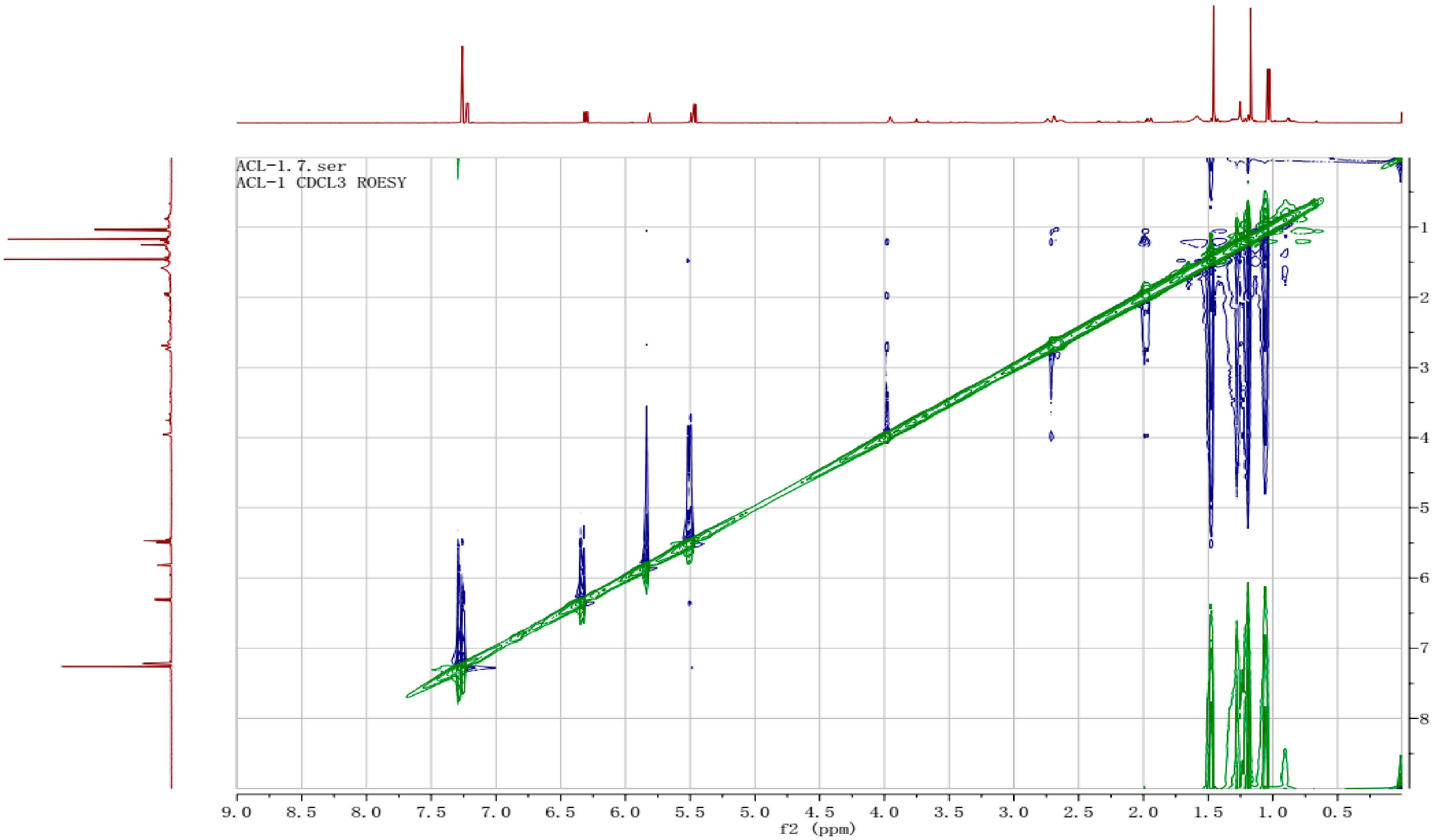

Figure S32. The HRESIMS and IR spectra of compound **4**

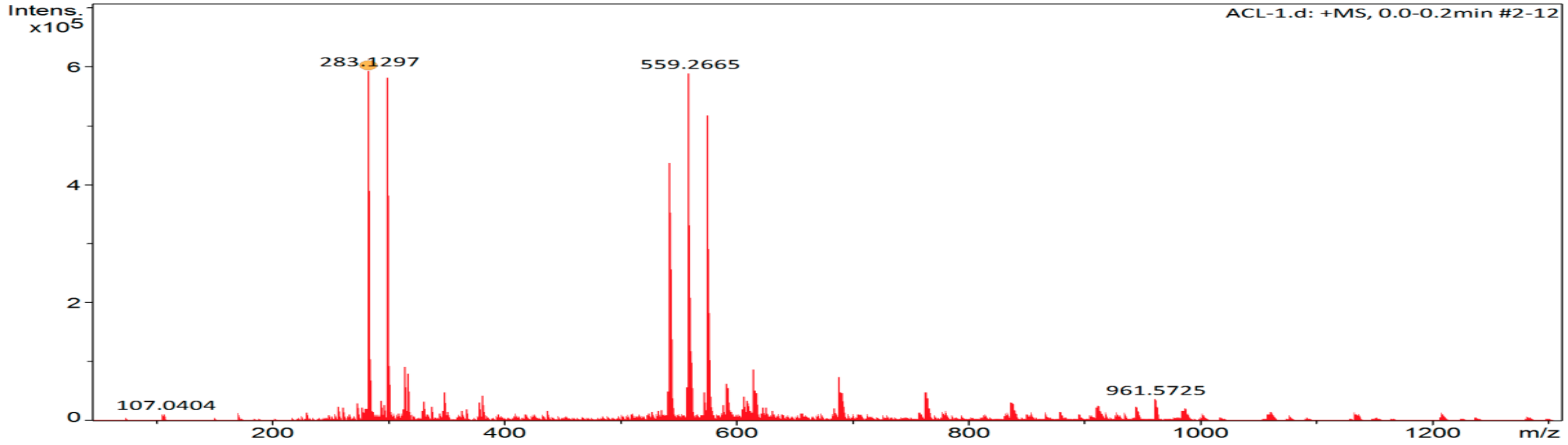

Sum Formula	Ion Formula	Meas. m/z	m/z	err [mDa]	err [ppm]
C16H20O3	C16H20NaO3	283.1297	283.1305	0.8	2.7

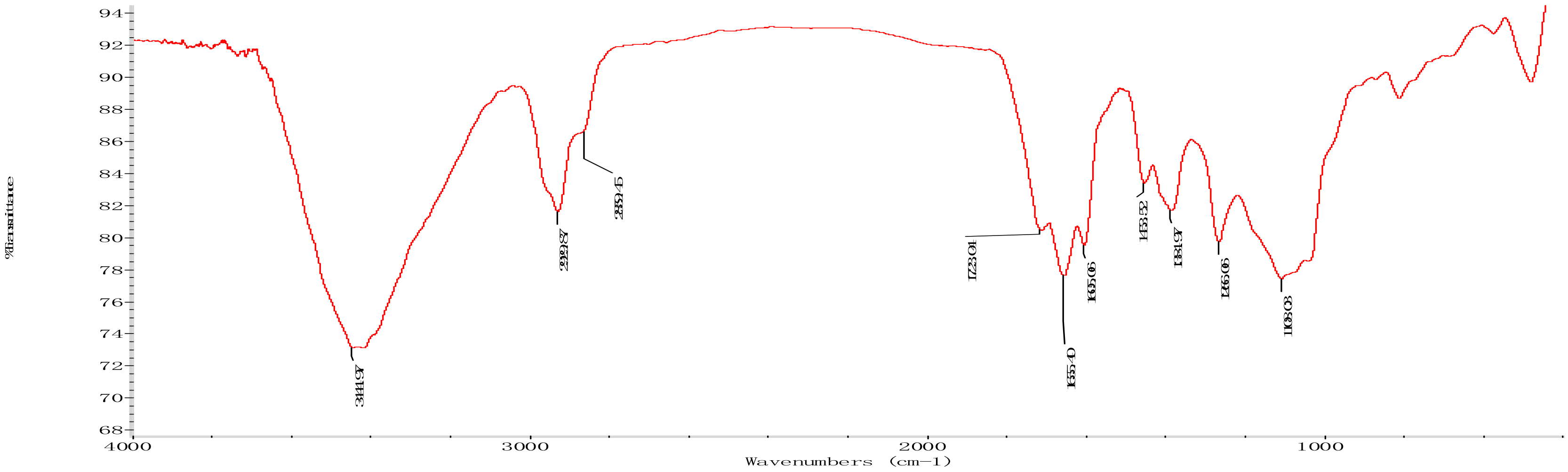

Figure S33. The ^{1}H NMR spectrum of compound **5** in $CDCl_3$

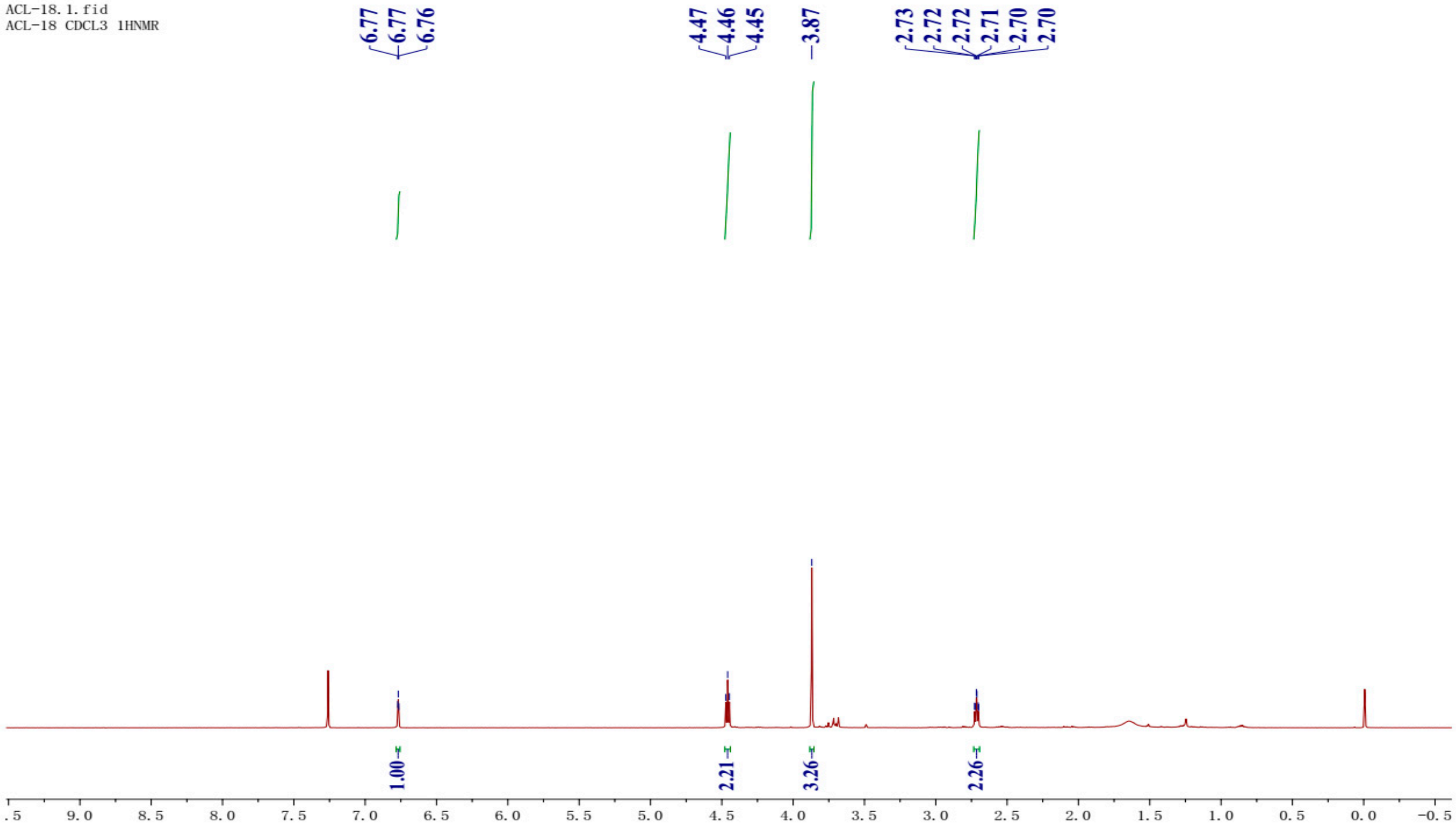

Figure S34. The ^{13}C-NMR spectrum of compound **5** in $CDCl_3$

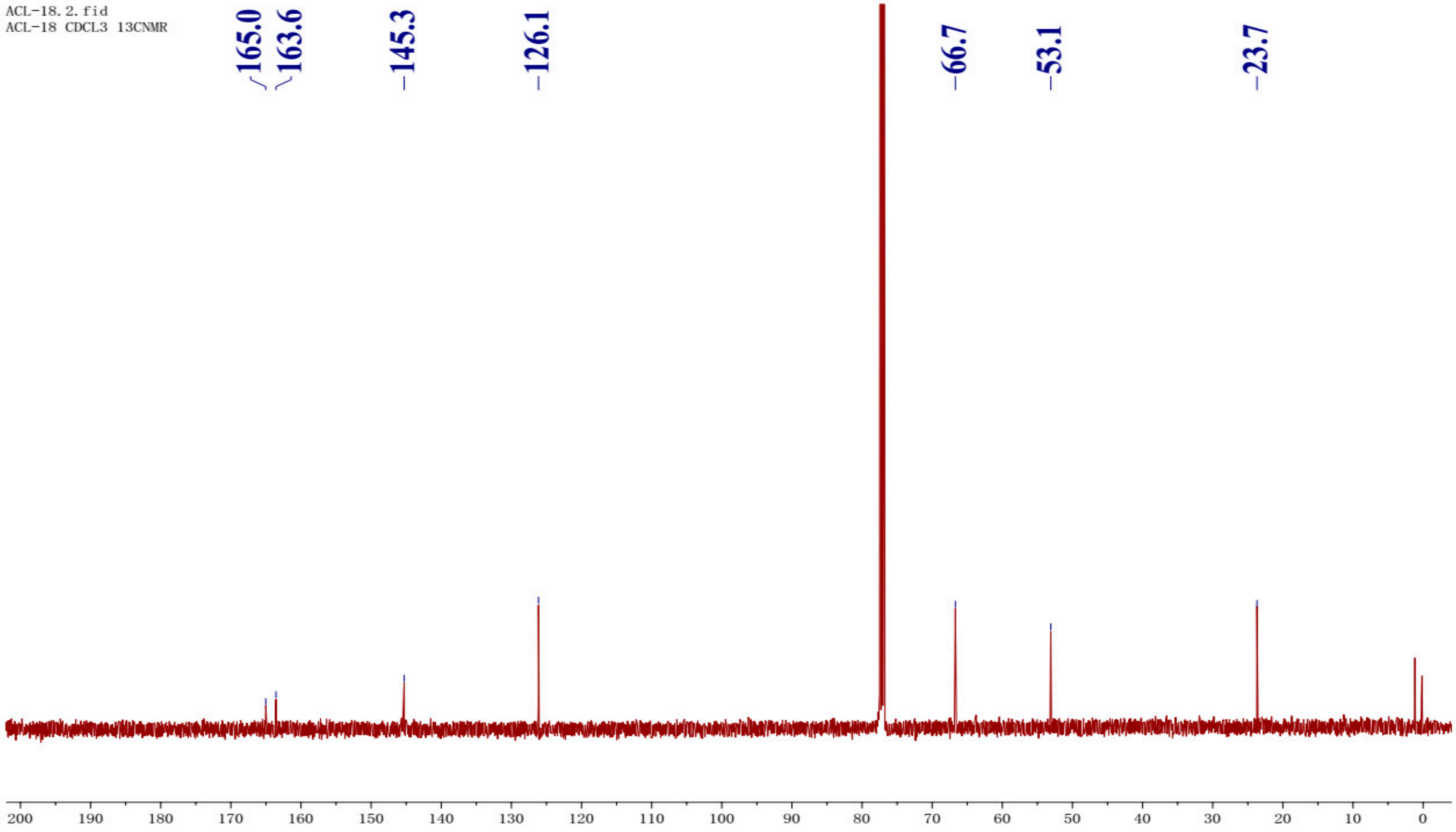

Figure S35. The DEPT spectrum of compound **5** in $CDCl_3$

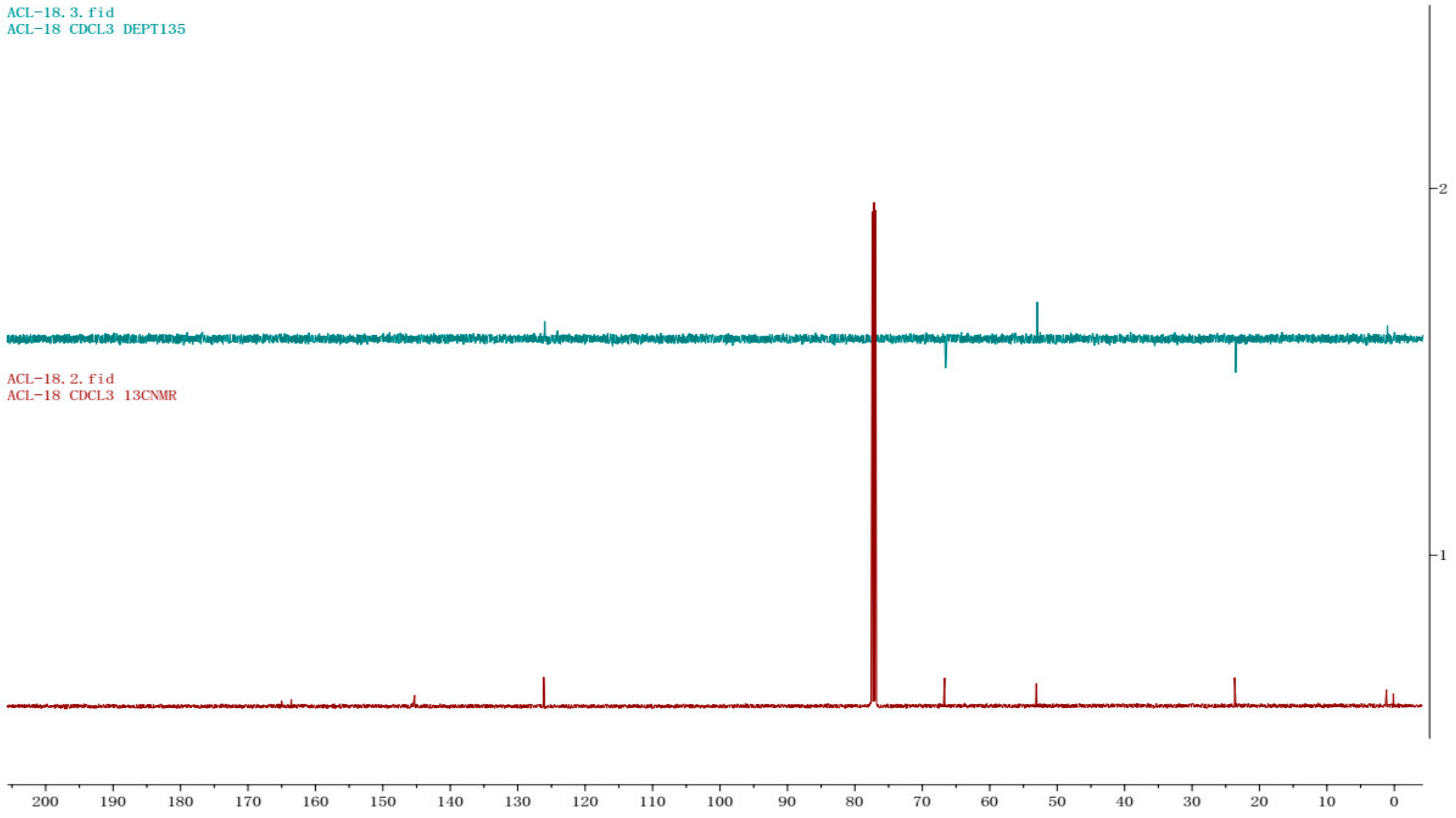

Figure S36. The HSQC spectrum of compound **5** in $CDCl_3$

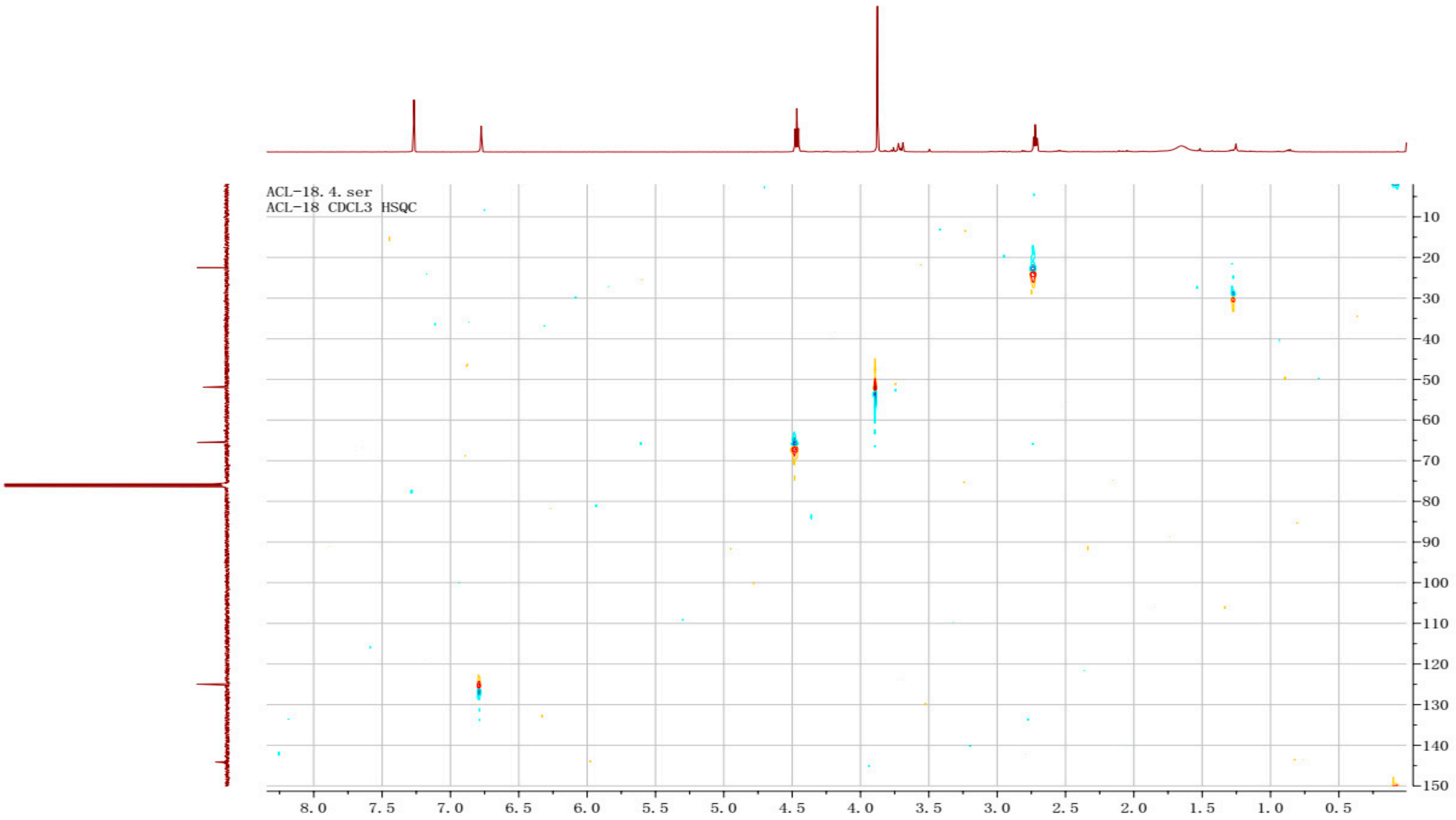

Figure S37. The ^1H-^1H COSY spectrum of compound **5** in $CDCl_3$

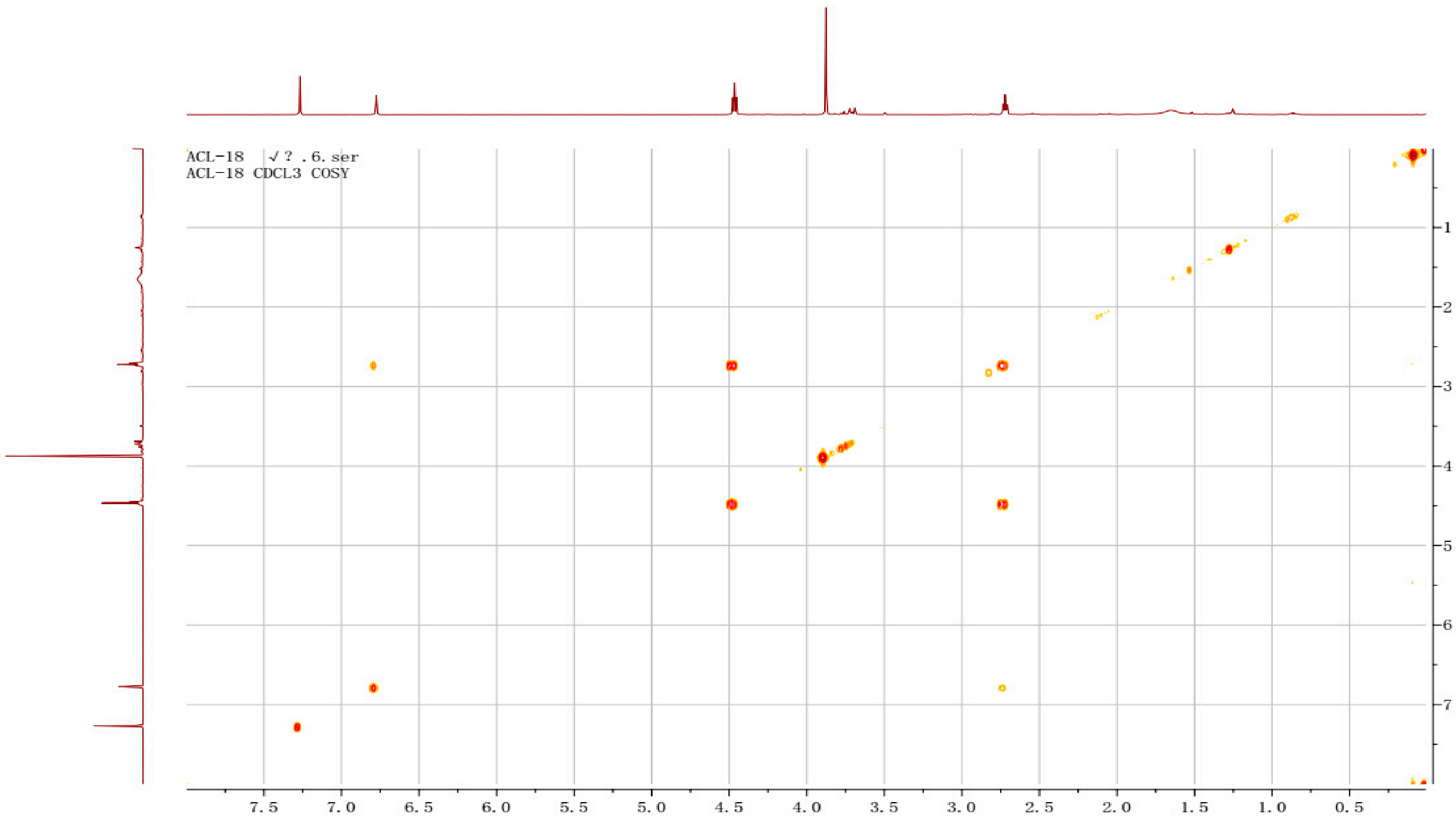

Figure S38. The HMBC spectrum of compound **5** in $CDCl_3$

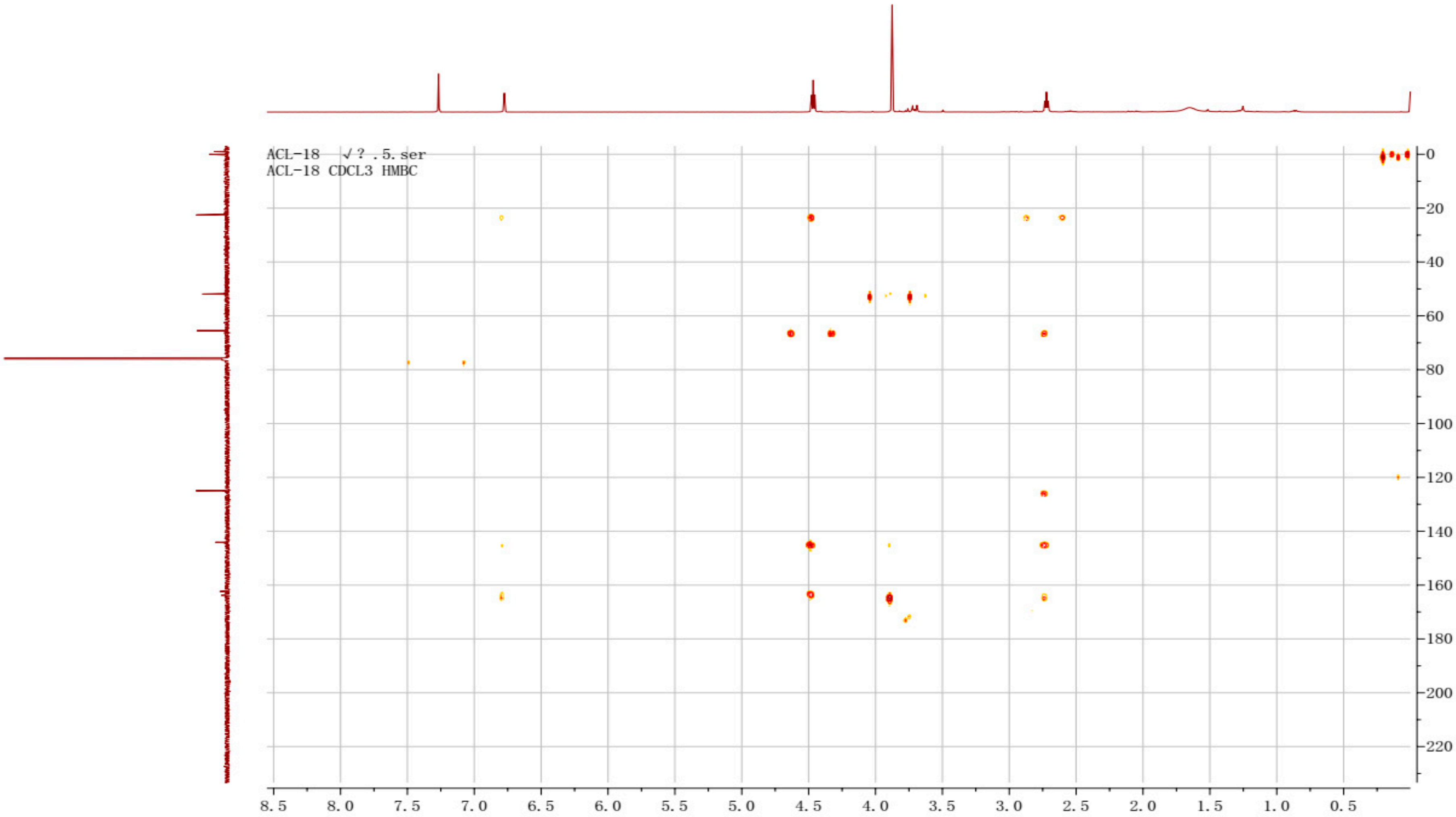

Figure S39. The HRESIMS and IR spectra of compound **5**

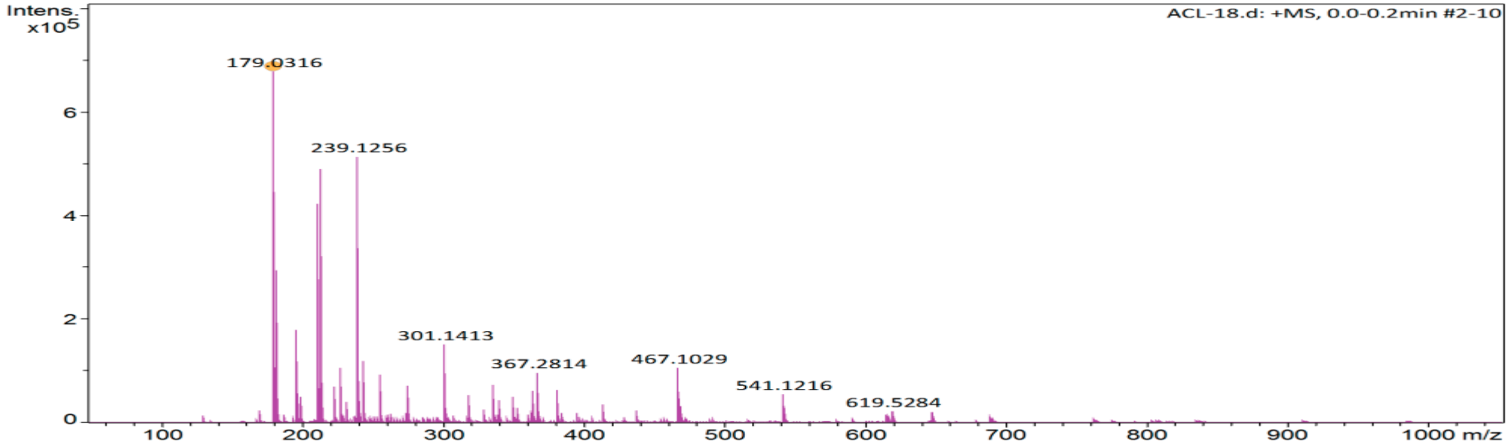

Sum Formula	Ion Formula	Meas. m/z	m/z	err [mDa]	err [ppm]
C7H8O4	C7H8NaO4	179.0316	179.0315	-0.1	-0.6

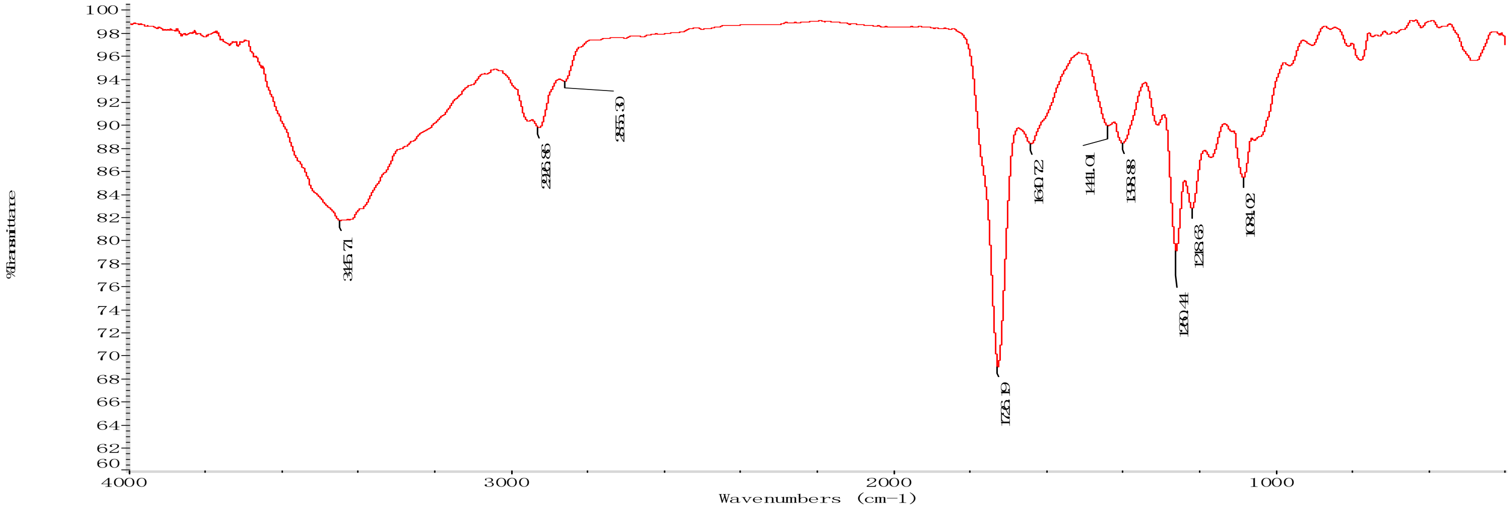

Supplement: Supplementary file 1 [file marinedrugs-16-00468-s001.pdf]
